# Processing and Characterization of UV Irradiated HDPE/POSS Fibers

**DOI:** 10.3390/nano13243131

**Published:** 2023-12-13

**Authors:** Ezgi Biçer, Mehmet Kodal, Güralp Özkoç

**Affiliations:** 1 Department of Chemical Engineering, Kocaeli University, 41001 Kocaeli, Turkey; ezgibicer39@gmail.com; 2 Nanotechnology Research and Application Center, Sabancı University, 34956 Istanbul, Turkey; guralp.ozkoc@istinye.edu.tr; 3 Department of Chemistry, Istinye University, 34396 Istanbul, Turkey; 4 Xplore Instruments B.V., 6135 KT Sittard, The Netherlands

**Keywords:** high-density polyethylene fiber, octavinyl POSS, methacryl POSS, crystallographic properties, mechanical properties

## Abstract

High-performance polyethylene fibers, renowned for their superior attributes encompassing a high strength, modulus, and lightness, are conventionally manufactured through the gel spinning method. However, this method is encumbered by several drawbacks, including the requisite application of a separate process to eliminate solvents from the fibers and the utilization of chemicals deleterious to both the environment and human health. Alternatively, the adoption of the melt spinning method presents a cleaner and environmentally friendly approach to attain high-performance fibers. In the present investigation, high-density polyethylene (HDPE) fibers were produced employing the melt spinning method. After the spinning process, strategic orientation procedures were implemented to enhance the crystallinity of the spun fibers. As a concluding step, seeking to elevate the overall performance of the oriented spun HDPE fibers, a cross-linking treatment was applied via UV irradiation. Notably, this study pioneers the incorporation of polyhedral oligomeric silsesquioxane (POSS) hybrid nanoparticles into HDPE during melt spinning, presenting a novel advancement aimed at further enhancing the mechanical properties of oriented HDPE fibers during UV irradiation. For this purpose, two distinct types of POSS, namely octavinyl POSS (OVPOSS) and methacryl POSS (MACPOSS), both having unsaturated double bonds capable of participating in the network structure of oriented HDPE spun during UV cross-linking, were used. The thermal, morphological, and mechanical properties, as well as the crystal structure of samples with and without POSS molecules, were investigated. The mechanical properties of the fibers exhibited higher values in the presence of OVPOSS. The incorporation of OVPOSS and MACPOSS resulted in a noteworthy improvement in the material’s tensile strength, exhibiting a marked increase of 12.5 and 70.8%, respectively. This improvement can be attributed to the more homogeneous dispersion of OVPOSS in HDPE, actively participating in the three-dimensional network structure. After orientation and UV irradiation, the tensile strength of HDPE fibers incorporating OVPOSS increased to 293 MPa, accompanied by a concurrent increase in the modulus to 2.8 GPa. The addition of POSS nanoparticles thus yielded a substantial improvement in the overall performance of HDPE fibers.

## 1. Introduction

Polyethylene (PE) fibers distinguish themselves from conventional counterparts by virtue of their exceptional properties, notably encompassing elevated strength and commendable chemical resistance. They have a wide range of uses in many fields, such as ballistic protectors, sports equipment, and maritime, aviation, and medical supplies [1]. Various techniques have been developed to produce high-modulus fibers from linear polyethylene [2]. These techniques include solid-state extrusion [3,4], hydrostatic extrusion [5], flow-induced crystallization [6], gel spinning followed by hot drawing [7,8], drawing of single crystal mats [9], drawing of pure polyethylene [10], and swell drawing [11,12,13]. The electrospinning method used in fiber production is not preferred in obtaining high-performance fibers. The electrospinning is complicated by its poor solubility in commonly used polar solvents and the need to raise and control process temperatures [14,15]. It has been stated in the literature that PE fibers produced by electrospinning do not exhibit significant mechanical properties [14]. Post-spinning orientation, as is the case with gel-spun PE fibers, is not suitable for electrospun fibers because they are not quickly spooled and reprocessed into continuous fibers. As a result, efforts to improve the fiber mechanical properties of electrospun PE by post-processing have been limited. Gel spinning is one of the most frequently used methods in the production of high-performance fibers. On the other hand, the gel spinning method has several disadvantages, such as the need for a separate process to remove the solvents from the fibers and the production using chemicals that are harmful to the environment and human health [16,17]. Alternatively, melt spinning stands as the prevalent method for the production of PE fibers, offering a solvent-free and ecologically conscientious process distinguished by its cleanliness and environmental friendliness. Therefore, melt spinning is an easy and productive approach compared to other spinning techniques, such as dry spinning and wet spinning [17].

Controlling fiber formation provides an opportunity to attain tunable properties in fibers [11]. Furthermore, post-treatments and the incorporation of polymer additives play pivotal roles in modifying the internal structure of the fibers. There are many studies in the literature using additives to improve the mechanical properties of polyethylene fibers. For instance, high-strength fibers obtained from high-density polyethylene (HDPE) and organoclay (Clayton HY) were examined in the study of Chantrakul and Amornsakchai [18]. The fibers were melted between 150 and 155 °C in a twin-screw extruder using the melt extrusion method. Fibers were obtained by adding organo-montmorillonite (OMMT) at 1, 3, and 6% by weight. Fibers with higher mechanical properties were obtained from organically modified montmorillonite spun composite fibers compared to HDPE fibers. The authors found the highest modulus and tensile strength to be 38 GPa and 1.7 GPa, respectively. OMMT has been found to allow high draw rates of the fiber and no agglomeration. Yeh et al. investigated the effect of functionalized nanoalumina fillers on the ultra- and superior tensile properties of high-molecular-weight polyethylene composite fibers [19]. Functionalized nanoalumina was added to ultra-high-molecular-weight polyethylene (UHMWPE) resin and its properties were examined. The authors observed that UHMWPE fibers prepared with functionalized nanoalumina had a tensile strength value of 6.4 GPa, 2.4 times higher than pure UHMWPE. It was determined that the functionalized nanoalumina was finely dispersed in polyethylene, thus increasing the specific surface area. As a result, crystallinity increases by creating more efficient areas for nucleation. In another study by Yeh et al., attapulgite (ATP) clay and modified attapulgite were used to improve UHMWPE’s ultradrawing and tenacity properties [20]. The acid-treated fibers exhibited a lower melting temperature and percent crystallinity than those with unmodified clay fibers. The authors stated that the drawing, orientation, and tensile test results of modified attapulgite fibers give better results compared to unmodified fiber.

In recent years, polyhedral oligomeric silsesquioxanes (POSSs) have garnered attention as alternative, next-generation, and environmentally friendly nano-additives for polymers. These materials contribute to enhanced flexible physical and chemical properties while exhibiting economic viability on an industrial scale. The general formula for a typical POSS is (RSiO_1.5_)_n_, where n is greater than 4 and mostly 8. Three bonds of Si are bonded into the cage, whereas the other bond can be attached to any functional group (R); therefore, many types of POSS can be synthesized [21,22]. Structurally, they are lattice-shaped molecules with different functional groups, such as vinyl, acrylate, methacrylate, epoxy, anhydride, hydroxyl, etc., where these chemical groups determine both the compatibility and reactivity of the nanoparticles [23]. POSS structures can be precisely functionalized with reactive groups for grafting, polymerization, and other transformations, a very popular strategy for creating nanometer-scale blocks in a variety of applications. Unlike conventional fillers, POSS molecules serve versatile purposes, such as nanoreinforcements [24], compatibilizers [25], flame retardants, cross-linkers [26], and even as a coagent for cross-linking PE [27].

In the literature, there are studies using POSS molecules for the production of polymer fibers [24,28,29]. For instance, Cozza et al. produced electrospun nanofibers based on poly(butylene terephthalate) (PBT) and POSS with different loading levels [28]. The authors observed that silsesquioxane molecules had a micrometric distribution and improved nanofiber alignment. In addition, the presence of POSS did not change the crystal structure formation of PBT macromolecules during the cold crystallization of nanofibers. In another study by Şirin et al., the effect of the POSS type and loading rate on melt-spun poly(ethylene terephthalate) (PET)-based composite fibers was investigated [24]. In this study, trisilanol isobutyl (TPOSS), aminopropyl isobutyl (APOSS), and glycidyl isobutyl were used as POSS types. POSSs were found to exhibit nucleating agent behavior. The highest %-crystallinity value was obtained in the presence of TPOSS. It also showed a higher birefringence value, indicating the overall orientation in the fiber, leading to lower thermal shrinkage. In a study by Tutak and Doğan, antimicrobial polypropylene (PP) fibers were produced using POSS nanoparticles during melt spinning [29]. For the modification of PP fiber, octa-ammonium POSS (QA-POSS) was used in three different concentrations: 0.5% and 1 and 3 wt%. It was concluded that as the amount of QA-POSS increased, the tensile strength and elongation at break values decreased. Thermal properties tend to drop similarly. The melting point and percentage of crystallinity of modified PP fibers decreased as the amount of QA-POSS was increased.

To enhance the mechanical and thermal properties of polyethylene (PE) fibers, the widespread adoption of cross-linking methodologies is employed. Cross-linking of PE fibers is conducted through different approaches, such as gamma or electron beam irradiation, and ultraviolet (UV), silanization, and peroxide-initiated methods. In the investigation conducted by Boer and Pennings, the impact of γ irradiation on the mechanical properties of ultra-high-strength polyethylene fibers was examined. The radiation dose was systematically varied within the range of 7 to 91 kGy. The authors observed an incremental rise in gel content with an increasing radiation dose, reaching a maximum of 85%. Concurrently, the tensile strength at break exhibited a reduction of approximately 40% [30]. In another study, Gao et al. examined the effects of different irradiation environments on the mechanical and structural properties of cross-linked UHMWPE fibers. Dyneema SK66 obtained from DSM was used as the fiber type. Within the scope of the study, the gel fraction, swelling ratios, and mechanical properties were investigated. Samples were irradiated in air, vacuum, and acetylene environments. While the gel fraction was 83% in acetylene, it was 44% in the vacuum and 36% in the air. The gel fraction was the highest due to the C≡C structure of acetylene. While the tensile strength decreased depending on the amount of the absorbed dose, the modulus value increased [31]. If the cross-linking process is effectively executed, it results in the establishment of a durable network characterized by covalent bonds. This network exhibits heightened resistance to external mechanical stress and thermal treatment in comparison to the original, non-crosslinked structure [32]. Recent attempts to cross-link drawn polyethylene via gamma or electron beam irradiation proved unsuccessful. Subsequently, UV irradiation was explored as an alternative technique, postulating that subjecting fibers to less energetic radiation relative to gamma or electron beam irradiation might mitigate the scission process of tensioned chains resulting from drawing. This method yielded notable improvements in creep behavior and enhanced thermal resistance through cross-linking, particularly when a coagent was concurrently present [32]. Therefore, UV irradiation has emerged as a viable method for instigating cross-linking in polyethylene.

POSS molecules can be used to improve the properties of cross-linked PE fibers. While POSSs provide cross-linking ability to fibers, they also create stronger cross-linking with their rigid cage structure. Thus, it reinforces the fibers at the molecular level. There are studies examining the effects of the radiation dose and environment on mechanical properties in the cross-linking of polyethylene. In this study, high-density polyethylene (HDPE) fibers with excellent mechanical and chemical properties were produced via the melt spinning method. After that, to improve the crystallinity of fibers, the orientation of fibers was carried out. Moreover, these fibers were cross-linked through UV irradiation to improve mechanical properties. The aim of this study was to obtain HDPE fibers with outstanding properties. For this purpose, POSS nanoparticles were incorporated into the HDPE during the melt blending method. The effect of POSS molecules on the properties of HDPE fibers oriented and UV cross-linked was investigated for the first time in the literature. Octavinyl POSS, with unsaturated groups, and methacryl POSS having both unsaturated and acrylic functional groups were used as POSS types. The logic behind the selection of these POSS nanoparticles is that they can participate in the cross-linking mechanism of HDPE fibers during UV irradiation. Therefore, they can contribute to the overall performance of PE fibers. For instance, increased crystallinity observed in both spun and oriented high-density polyethylene (HDPE) upon the incorporation of OVPOSS and MACPOSS led to a significant enhancement in the modulus. It was observed that MACPOSS displayed a heterogeneous dispersion, whereas OVPOSS demonstrated a homogeneous dispersion. In addition, they contribute to an environmentally friendly fiber spinning process since this process with POSS nanoparticles does not require any solvent.

## 2. Materials and Methods

### 2.1. Materials

**Scheme 1:**
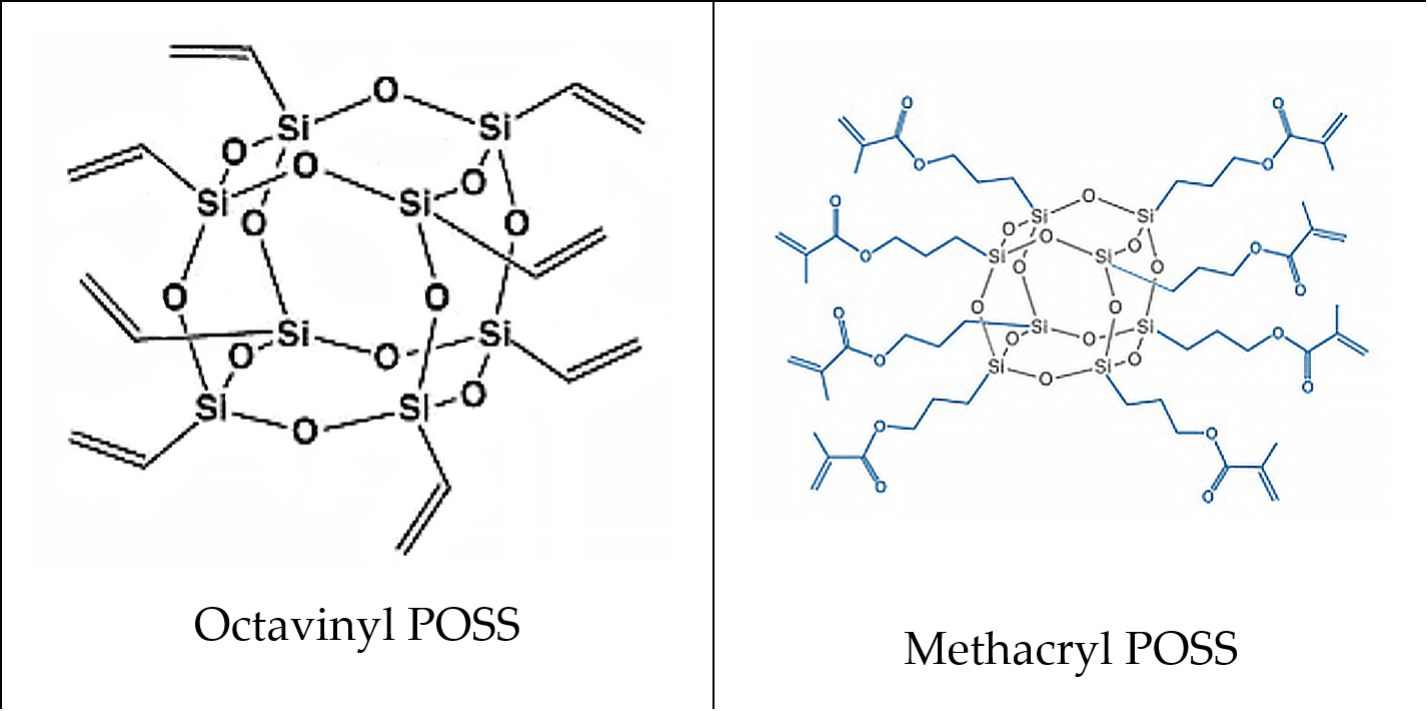
The chemical structure of POSS types.

The PETILEN YY I668(UV) high-density polyethylene was purchased from PETKIM Petrochemical Holding Inc., Türkiye. According to the information supplied by the manufacturer, melting temperature (T_m_), density, and melt flow index (MFI) values of the HDPE were 134 °C, 0.965 g/cm^3^, and 5.5 g/10 min (190 °C/2.16 kg), respectively. OVPOSS and MACPOSS were obtained from Hybrid Plastics Inc., USA. OVPOSS, a white crystalline powder, is soluble in tetrahydrofuran (THF) and chloroform, having a molecular weight of 632.31 Da. MACPOSS is a yellowish transparent liquid thermally stable up to 386 °C, soluble in THF, ethanol, and chloroform. The chemical structures of the aforementioned POSS types are illustrated in Scheme 1.

### 2.2. Fiber Spinning and UV Cross-Linking

**Scheme 2:**
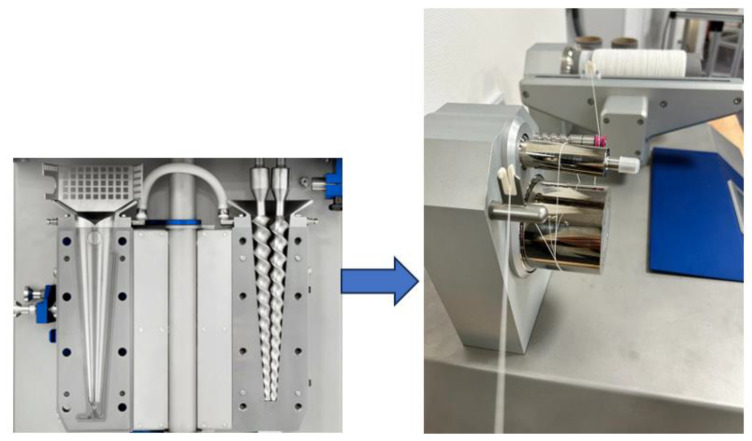
MC 15 HT twin-screw micro-compounder and fiber spinning unit (Xplore Instruments, Sittard, the Netherlands).

HDPE/POSS samples were prepared through melt compounding utilizing an MC 15 HT laboratory scale twin-screw micro-compounder (Xplore Instruments B.V., Sittard, The Netherlands). The process parameters were set with a screw speed of 50 rpm and a barrel temperature of 145 °C, and the compounding time was 3 min. The content of POSSs was kept constant at 4 phr. The filament samples were produced with an Xplore model monofilament spinning unit having a nozzle diameter of 1.25 mm at a controlled temperature, directly coupled with the MC 15 HT (Scheme 2). The fiber spinning process was conducted at a controlled temperature, with a winding speed of 50 m/min and a winding width of 120 mm. The compositions of the fibers investigated in this study are given in Table 1. Subsequently, the spun fiber samples underwent a 500% orientation process facilitated by a drawing unit. Cross-linking was achieved by exposing the fibers to UV radiation for a duration of 10 min in a cabinet equipped with four lamps, each with a power of 400 W.

### 2.3. Characterization

#### 2.3.1. Fourier Transform Infrared Spectroscopy Analyses

Chemical structure properties of the fibers were analyzed with Fourier transform infrared spectroscopy (Perkin Elmer Inc., Waltham, MA, USA) using a Spectrum 100 FTIR device equipped with an ATR unit. The FTIR spectra in the range of 650–4000 cm^−1^ were collected.

#### 2.3.2. Gel Content Measurements

To measure the gel content, cross-linked fiber specimens underwent Soxhlet extraction in p-xylene following the procedure outlined in accordance with ASTM D 2765-16 standard specifications [33]. Approximately 0.5 g of the sample was arranged in small fragments within a stainless-steel wire mesh and subjected to a 24 h extraction in p-xylene at 140 °C. After extraction, the swollen samples were weighed. Subsequently, the samples were cleaned with acetone, subjected to drying in a vacuum oven at 100 °C until a constant weight was achieved, and then re-weighed. The percentage of gel content is calculated as the difference between W_s_, denoting the weight of the initially swollen sample, and W_d_, representing the weight of the dried gel sample. This difference is then proportioned to the original polymer weight W_o_. The percentage of gel content is shown in Equation (1):

(1) $$\mathrm{Gel}\;\mathrm{content}\;\left(\%\right)=\;\frac{\left(W_{s}-W_{d}\right)}{W_{o}}\times 100$$

#### 2.3.3. Mechanical Properties

Tensile testing was executed on a subset of 10 randomly selected samples, each conditioned at room temperature for a duration of 24 h. Average fiber diameters were ascertained for individual filament samples through examination with a Nikon model polarized optical microscope. An Instron Universal Testing Machine with a 5 kN load cell was employed for tensile testing. The specimens were subjected to testing over a fixed gauge length of 50 mm, with a consistent crosshead speed of 50 mm/min.

#### 2.3.4. Morphological Analyses

Fiber surfaces were examined with an optical microscope. To ascertain the average diameter thickness, a minimum of 50 measurements were taken from each mixture. Additionally, the melt crystallization behavior of the compounds during cooling was investigated employing a Nikon model polarized optical microscope (POM). Throughout the crystallization stage, the samples were heated to 150 °C with a heating rate of 30 °C/min. The thermal history was reset by maintaining the sample at 150 °C for 5 min, followed by cooling from 150 °C to 25 °C at a rate of 5 °C/min. Images capturing crystal formation were acquired between 130 °C and 120 °C during the cooling step.

The phase distributions and surface topographies of the fibers were elucidated through an analysis with a Nanosurf NaioAFM Atomic Force Microscope (AFM, Lisztar, Switzerland). AFM images were generated by scanning in the dynamic force mode using a silicon cantilever with a scanning speed of 2 s. The dimensions of the scanned images were 6 µm × 6 µm.

#### 2.3.5. X-ray Diffraction Analyses

X-ray diffraction (XRD) analyses were conducted on the fibers using a RIGAKU device, employing a scanning speed of 2°/min within the range of 0–60°. For the examination of samples, including POSS nanoparticles, the scanning range was set between 0 and 40° at a speed of 2°/min. CuKα radiation at 35 kV and 15 mA was utilized, with a 1.54 Angstrom X-ray source. The percent crystallinity was calculated using Equation (2) as follows:

(2) $$\mathrm{Crystallinity}\;\left(\%\right)=\;\;\frac{\mathrm{Overall}\;\mathrm{crystal}\;\mathrm{area}}{\mathrm{Crystal}\;\mathrm{and}\;\mathrm{amorphous}\;\mathrm{areas}\;\mathrm{in}\;\mathrm{total}\;}\;\times \;100$$

The Scherrer equation, denoted as Equation (3), was employed for the determination of crystal size [34]. In this equation, the variable “L” represents the crystal size. The X-ray wavelength (λ) is maintained as a constant at 1.54056 Å for CuKα, which is employed in the θ − 2θ Bragg–Brentano geometry [35]. The value of “k” is 0.94 [36], applicable to spherical crystals with cubic–metric lattice symmetry. The full-width half-maximum, B, was calculated in the radian angle.

(3) $$\mathrm{Scherrer}\;\mathrm{equation}\;\left(L\right)=\;\frac{k\lambda }{\mathrm{Bcos}\theta }$$

#### 2.3.6. Thermal Analyses

A differential scanning calorimeter (DSC, Mettler Toledo LLC., Columbus, OH, USA) was used to investigate the thermal behavior of spun and oriented fibers. A DSC analysis was carried out between 25 °C and 230 °C at a heating rate of 10 °C/min under a nitrogen atmosphere. The melting behavior of the samples was obtained from the DSC thermograms. The %-crystallinities of the samples, X_c_ (%), were determined from Equation (4):

(4) $$X_{c}\left(\%\right)=\frac{\Delta H_{m}}{{\Delta H*}_{m}\;(1-Ø)}\;\times \;100$$

where “∆H_m_” is the melting enthalpy (J/g), “∆H*_m_” is the heat of fusion for 100% crystalline HDPE, taken as 293 J/g [37], and Ø is the weight fraction of POSS in the samples.

To determine the thermal stability and ash content of the samples, thermal gravimetric analyses (TGAs) in the nitrogen atmosphere were carried out with a TA Instruments Thermal Analysis device. Samples were analyzed from 25 °C to 600 °C with a heating rate of 10 °C/min.

## 3. Results and Discussions

### 3.1. Chemical Properties and Gel Content of Samples

**Figure 1:**
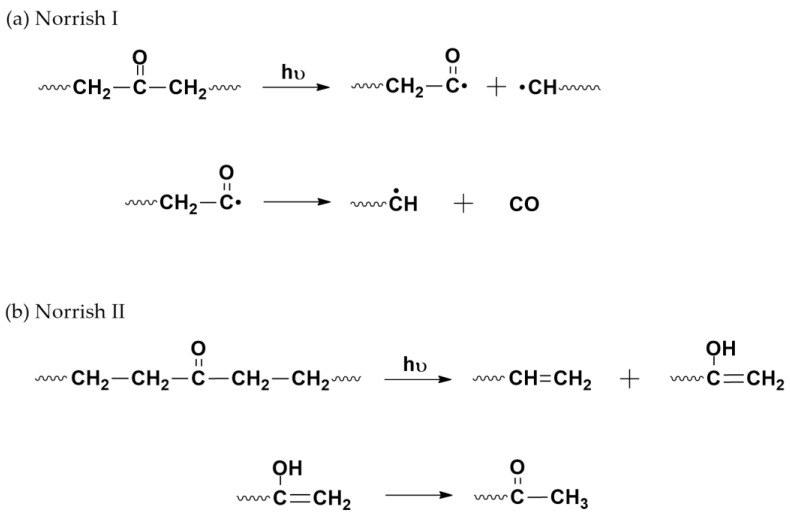
Norrish I and Norrish II degradation mechanisms: (**a**) Norrish I degradation can lead to chain scission or cross-linking; (**b**) Norrish II degradation leads to chain scission.

**Figure 2:**
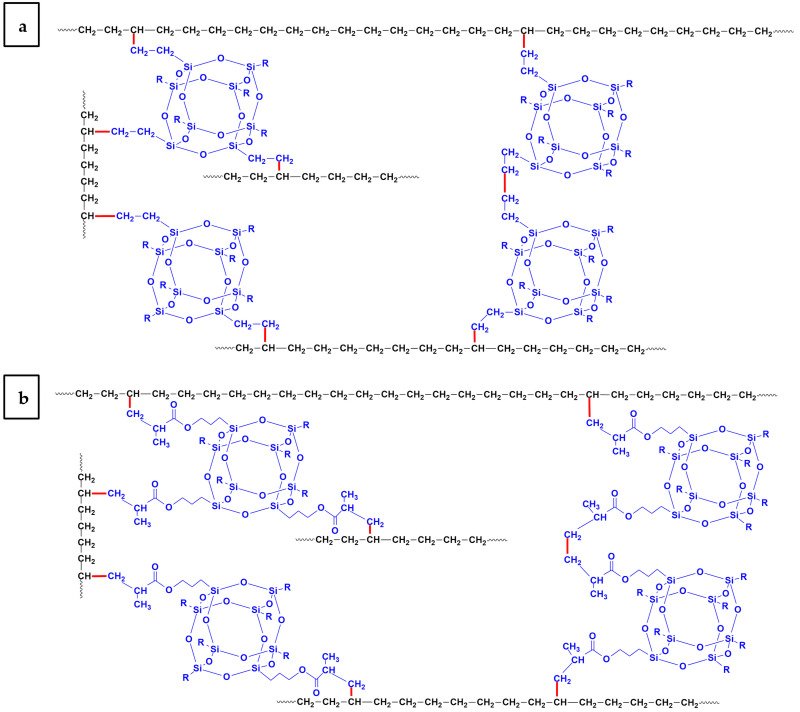
UV cross-linking reaction mechanism of HDPE fibers: (**a**) HDPE/OVPOSS, (**b**) HDPE/MACPOSS.

Decomposition reactions arise from the carbonyl group through Norrish type I and II reactions [38]. In instances where the degradation of carbonyl groups proceeds in accordance with the Norrish I reaction, resultant free radicals may initiate attacks on the polyolefin, leading to chain breaks (Figure 1a), culminating in termination either through cross-linking or chain scission. Conversely, if degradation proceeds in line with the Norrish II reaction, carbonyl groups and terminal vinyl groups are generated (as illustrated in Figure 1b), thereby causing chain breakage. Additionally, the carbonyl group formed exhibits a higher susceptibility to degradation. The photodegradation of polyethylene involves a competition between chain scission and cross-linking mechanisms [39]. Chain scission results in a reduction in the polymer’s molecular weight, while cross-linking enhances interaction between polymer chains, increasing its molecular weight. The photo-degradation of polyethylene yields three predominant functional groups, namely ketones, carboxylic acids, and vinyl groups. The formation of carbonyl and vinyl groups serves as a direct indicator of main chain cleavage [40]. The decomposition of polyethylene induces an increment in crystallinity, signifying chain cleavage. The resulting shorter chains, originating from cleavage, exhibit heightened mobility and facilitate enhanced crystallization, contributing to an associated increase in brittleness. The UV cross-linking reaction mechanisms for HDPE fibers, including octavinyl POSS and methacryl POSS, are depicted in Figure 2a and Figure 2b, respectively.

**Figure 3:**
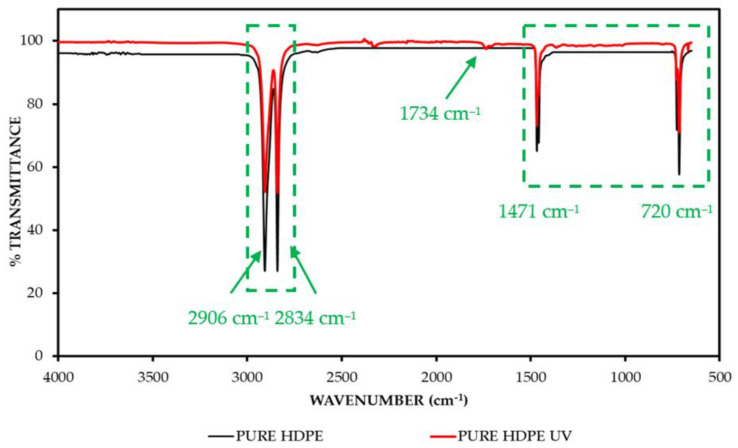
FTIR spectra of spun HDPE fiber and UV-irradiated HDPE fiber.

**Figure 4:**
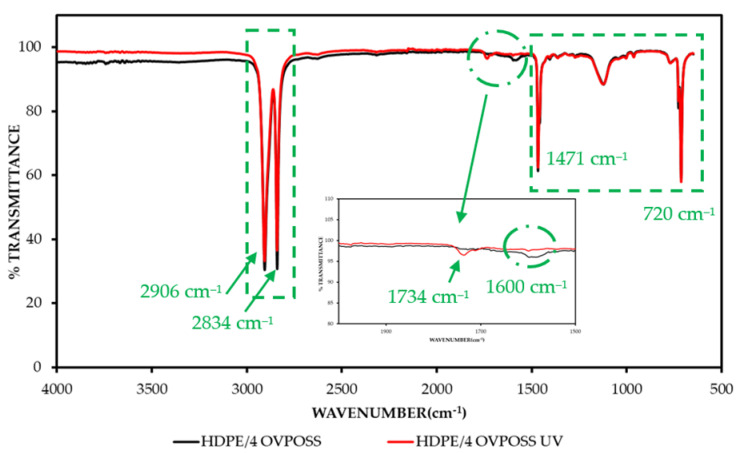
FTIR spectra of spun HDPE/OVPOSS fiber and UV-irradiated HDPE/OVPOSS fiber.

**Figure 5:**
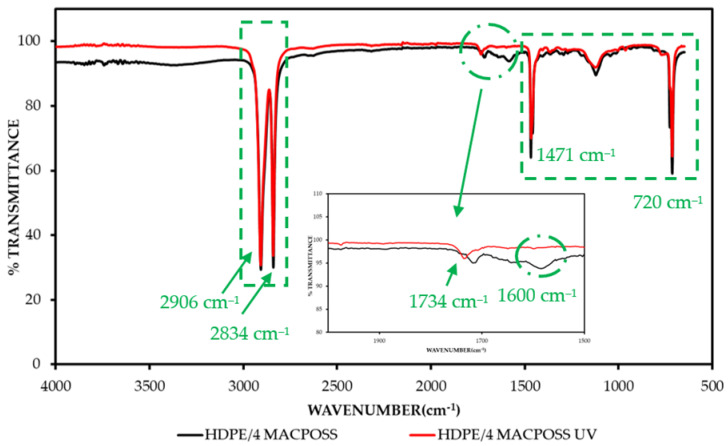
FTIR spectra of spun HDPE/MACPOSS fiber and UV-irradiated HDPE/MACPOSS fiber.

The ATR-FTIR spectra of spun and UV-irradiated samples are shown in Figure 3. The discernible bands at 2906, 2834, 1471, and 720 cm^−1^ align with the characteristic vibration modes of saturated methylene (-CH_2_) structures in polyethylene [41]. These absorption bands correspond to wagging, symmetric stretching, and asymmetric stretching vibration of (-CH_2_) groups [42]. Notably, a reduction in the intensity of the 2906, 2834, 1471, and 720 cm^−1^ wavenumbers is evident upon UV cross-linking, as illustrated in Figure 3, Figure 4 and Figure 5. This is much more prominent for HDPE samples after UV irradiation. The observed alterations in the FTIR spectra peaks for HDPE can be attributed to simultaneous chain scission with oxidation reactions and the UV-induced cross-linking process, manifesting as changes in the absorption band heights. At the same time, after UV treatment, peak formation was observed at the wave number of 1818–1750 cm^−1^ belonging to the carbonyl stretching (-C=O) groups. The peaks at 1718 cm^−1^ and 1734 cm^−1^ specifically denote the presence of a limited number of carbonyl structures, specifically ketones and aldehydes [43]. The formation of the peaks located at 1645 and 909 cm^−1^ indicates the formation of unsaturated vinyl groups. The absence of these peaks in UV cross-linked fiber samples suggests the non-formation of unsaturated vinyl groups. The disappearance of the characteristic stretching vibration peak located at 1600 cm^−1^ (C=C) related to OVPOSS and MACPOSS implies that POSSs are integrated into the structure via cross-linking [44]. Moreover, there is a lesser decrease in the intensity of saturated methylene (-CH_2_) groups in fibers containing OVPOSS and MACPOSS compared to pure HDPE. Furthermore, in MACPOSS crosslinked samples, the absorption band initially located at 1717 cm^−1^ shifted to 1734 cm^−1^, signifying another indicator of the chemical bonding of MACPOSS after UV irradiation. This showed that cross-linking reactions are more pronounced than chain scission reactions in the presence of POSS.

**Figure 6:**
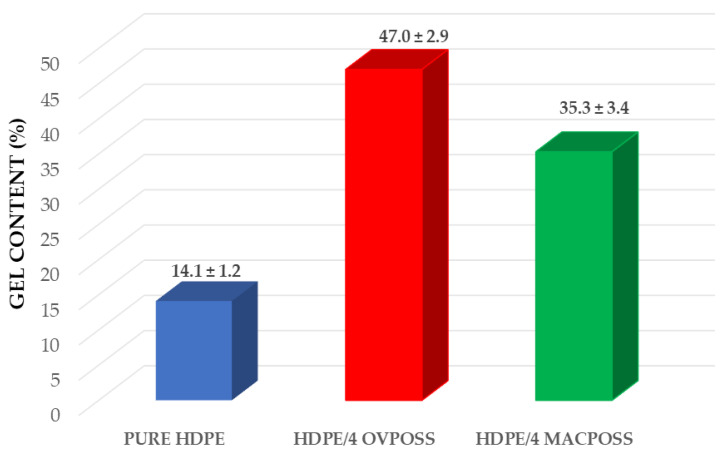
Gel content of the oriented HDPE/POSS fibers after UV irradiation.

The gel content, derived from the Soxhlet analysis of the cross-linked samples, is presented in Figure 6. HDPE fibers without POSSs exhibited a 14% gel content. Notably, an augmentation in gel content was evident with the incorporation of POSSs. Specifically, the percentage of gel increased to 47% with the addition of OVPOSS, while MACPOSS resulted in a gel content of 35%. Notably, OVPOSS demonstrated a more pronounced effect on cross-linking compared to MACPOSS, which can be attributed to more double bonds than MACPOSS. Therefore, the ability to participate in OVPOSS in the cross-linking mechanism of HDPE is more noticeable.

### 3.2. Mechanical Properties

**Figure 7:**
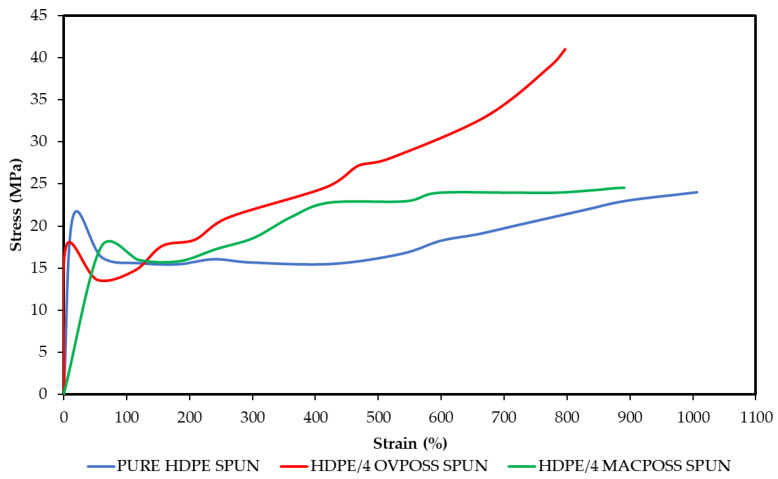
Stress–strain curves of spun HDPE and HDPE/POSS fibers.

**Figure 8:**
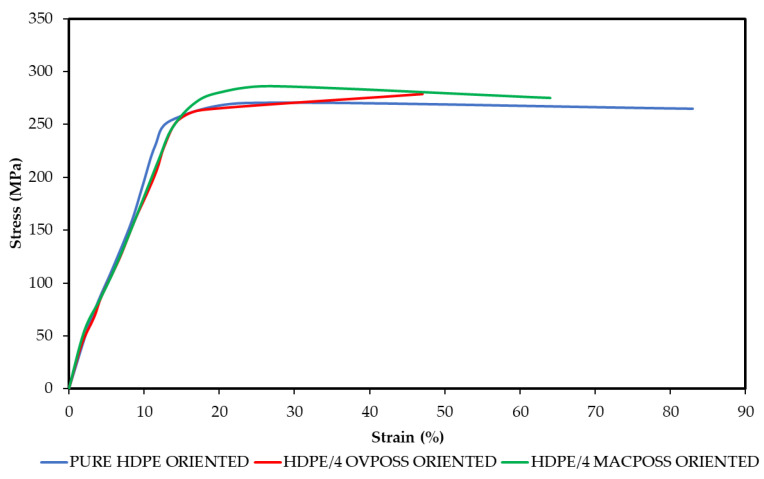
Stress–strain curves of oriented HDPE and HDPE/POSS fibers.

**Figure 9:**
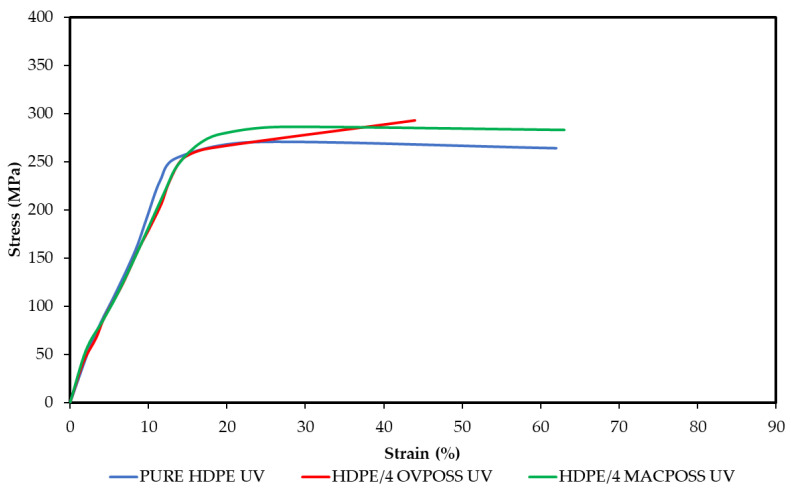
Stress–strain curves of oriented HDPE and HDPE/POSS fibers after UV irradiation.

The tensile test results of the samples can be seen in Figure 7, Figure 8 and Figure 9. Obtained values from tensile curves were tabulated in Table 2. Figure 7 shows the stress–strain curves of spun HDPE/POSS fibers. As seen in Figure 7, all the samples exhibited stress-induced crystallization after necking. POSS addition facilitated the reorganization of HDPE chains, which resulted in higher tensile strength values. This is much more prominent in the presence of OVPOSS nanoparticles. On the other hand, the elongation at break values of HDPE spun fibers, including POSS nanoparticles, exhibited lower values as a comparison with HDPE spun fibers. This was due to the rigid cage structure of POSS molecules, resulting in increased entanglement density. Therefore, the elongation at break values decreased in the presence of POSS molecules.

Tensile stress–strain curves of oriented HDPE/POSS fibers are shown in Figure 8. The necking behavior disappeared after orientation. Moreover, tensile strength significantly increased; however, elongation at break values decreased as expected. In addition, the slope of the curves notably enhanced after the orientation of the samples, regardless of the POSS type and POSS addition. All these findings can be attributed to the increased crystallinity of oriented HDPE fibers after the addition of POSSs, as discussed in the DSC part of the manuscript.

The tensile stress–strain curves of oriented and UV-irradiated HDPE/POSS fibers are illustrated in Figure 9. A noticeable enhancement in the tensile strength values of the samples is observed after UV irradiation. This improvement can be attributed to the formation of cross-linking and rigid structures induced by the presence of polyhedral oligomeric silsesquioxanes, resulting in an overall enhancement of mechanical properties. Furthermore, the percent elongation at break exhibited a decrease, indicative of the anticipated trend arising from the constraints imposed on the fiber structure due to cross-linking.

Tensile strength and elongation at break of the spun and oriented HDPE/POSS fibers are shown in Table 2. The tensile strength of the spun HDPE fibers without POSSs was found to be 24 MPa. Following orientation, the tensile strength values of the spun HDPE fibers experienced a tenfold increase, reaching 265 MPa. The elongation at break was observed to decrease from 1007% to 83% due to the orientation process, resulting in an improvement in crystallinity. The incorporation of OVPOSS led to an approximately 70% increase in the tensile strength of the HDPE spun fibers, while the addition of MACPOSS resulted in a strength value of 25 MPa for the spun fibers. The tensile strength values were further augmented with the orientation process. Notably, OVPOSS exhibited higher strength values compared to MACPOSS after orientation, indicative of enhanced interfacial interaction between OVPOSS and HDPE fibers, corroborated by morphology analyses. Moreover, the increased gel content in the presence of OVPOSS displayed superior mechanical properties. The elongation at break tended to decrease during orientation, and the addition of POSS nanoparticles into HDPE further reduced elongation at break values, attributed to the rigid inorganic silica structure of POSS molecules. Among the POSS-reinforced oriented HDPE fibers, the highest elongation at break was observed for HDPE/methacryl POSS fibers, owing to the higher content of flexible methylene groups in their side chains. Conversely, the silica groups of OVPOSS, directly attached to double bonds, introduced greater rigidity, resulting in increased chain entanglement in HDPE fibers and lower elongation at break values. Upon cross-linking with UV, tensile strength values increased to approximately 290 MPa. Compared to pure polyethylene fiber, POSS nanoparticles enhanced the mechanical properties, such as tensile strength, by participating in the cross-linking mechanism of HDPE. However, the elongation at break values exhibited a declining trend due to the resistance of the cross-links to flow.

Conversely, the increased chain entanglement of both spun and oriented HDPE with the addition of OVPOSS tremendously improved the modulus. Furthermore, the enhanced modulus values associated with OVPOSS were attributed to the heightened crystallinity of HDPE, as extensively discussed in the DSC and XRD sections of the manuscript. The incorporation of methacryl POSS into HDPE also contributed to the enhancement of the elastic modulus in spun and oriented HDPE fibers. However, in comparison to OVPOSS fibers, the modulus values in the presence of MACPOSS were not as elevated; however, it is still higher than cross-linked and oriented HDPE fibers. This discrepancy can be attributed to the lack of favorable interface formation, as indicated in the morphological analysis, resulting in a comparatively less pronounced improvement in mechanical properties. In addition, as seen in Table 2, energy at break, or in other words, tensile toughness values, of all samples decreased after the orientation and UV irradiation process as expected. This decrement in tensile toughness values was more prominent in oriented HDPE spun fibers in the presence of POSS molecules, which was due to the enhanced crystallinity. Similarly, higher crystallinity and cross-link density resulting from UV irradiation decreased the energy at break values of all samples compared to energy at break values of HDPE spun fibers with and without POSSs.

### 3.3. Morphology

**Figure 10:**
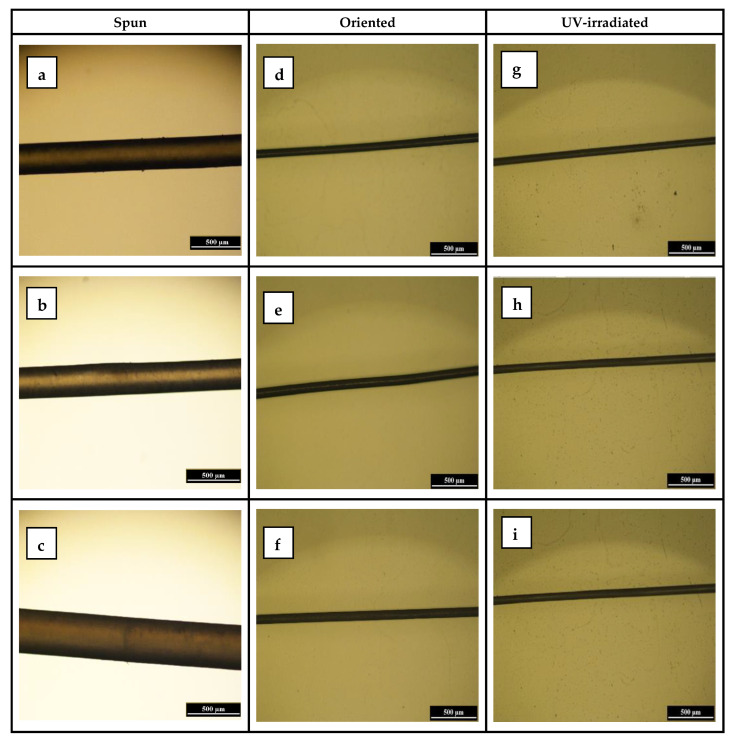
Optical microscope images of the HDPE fibers: (**a**) spun pure HDPE, (**b**) spun HDPE/OVPOSS, (**c**) spun HDPE/MACPOSS, (**d**) oriented pure HDPE, (**e**) oriented HDPE/OVPOSS, (**f**) oriented HDPE/MACPOS, (**g**) pure HDPE after UV, (**h**) HDPE/OVPOSS after UV, (**i**) HDPE/MACPOSS after UV.

Optical microscope images depicting spun and oriented HDPE fibers are presented in Figure 10, revealing uniformly smooth surfaces across all fibers. The addition of MACPOSS is associated with an observable increase in fiber diameter, whereas the introduction of OVPOSS yields a diameter comparable to that of the pure HDPE spun fiber. The average fiber diameters of spun, oriented, and UV-irradiated HDPE/POSS fibers are demonstrated in Table 3. The pure HDPE spun fiber exhibited an average fiber diameter of 237 ± 22 µm, with the addition of MACPOSS resulting in a discernible increase to 346 ± 27 µm. Conversely, the addition of OVPOSS did not significantly alter the fiber diameter. The orientation process led to a reduction in fiber diameters, as expected. OVPOSS, in particular, facilitated the production of finer fibers compared to MACPOSS. The mean fiber diameter for the oriented HDPE spun fiber was measured at 92 ± 3 µm. In the case of oriented spun fibers containing OVPOSS, the mean diameter decreased to 78 ± 8 µm. Additionally, the oriented fiber diameter gradually decreased with the addition of MACPOSS. Relative to the spun HDPE fiber, fiber diameters showed a reduction of 62% due to orientation, while this reduction was 67% and 73% in the presence of OVPOSS and MACPOSS, respectively. The orientation-induced decrease in fiber diameters is attributed to the alignment of molecular chains in a more parallel orientation, leading to a reduction in free volume and internal arrangement. Notably, no significant changes in diameters were observed after UV treatment.

**Figure 11:**
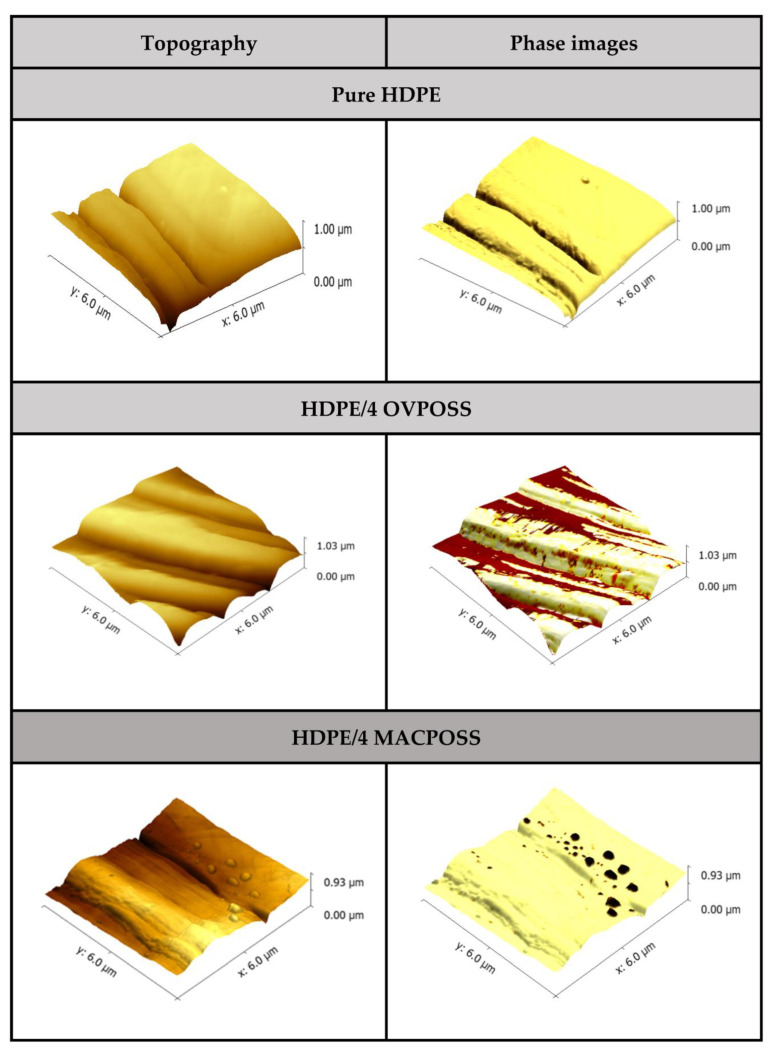
Topography and phase images of the spun HDPE and HDPE/POSS fibers.

An atomic force microscope was used to examine the topography and phase images of the samples, employing the dynamic mode. Figure 11 shows the topography images of spun fibers alongside phase distributions. The addition of OVPOSS distinctly alters the fibrillar structure of HDPE spun fibers. Conversely, the incorporation of MACPOSS leads to the formation of fibers with varying diameters. Upon examination of the phase images, a homogeneous dispersion of OVPOSS is evident, attributed to the compatibility of nonpolar OVPOSS with nonpolar HDPE. In contrast, MACPOSS exhibits a micron-scale distribution, displaying some coarse dispersion within the HDPE matrix. This disparity is attributed to the polarity differences between polar MACPOSS and nonpolar HDPE.

**Figure 12:**
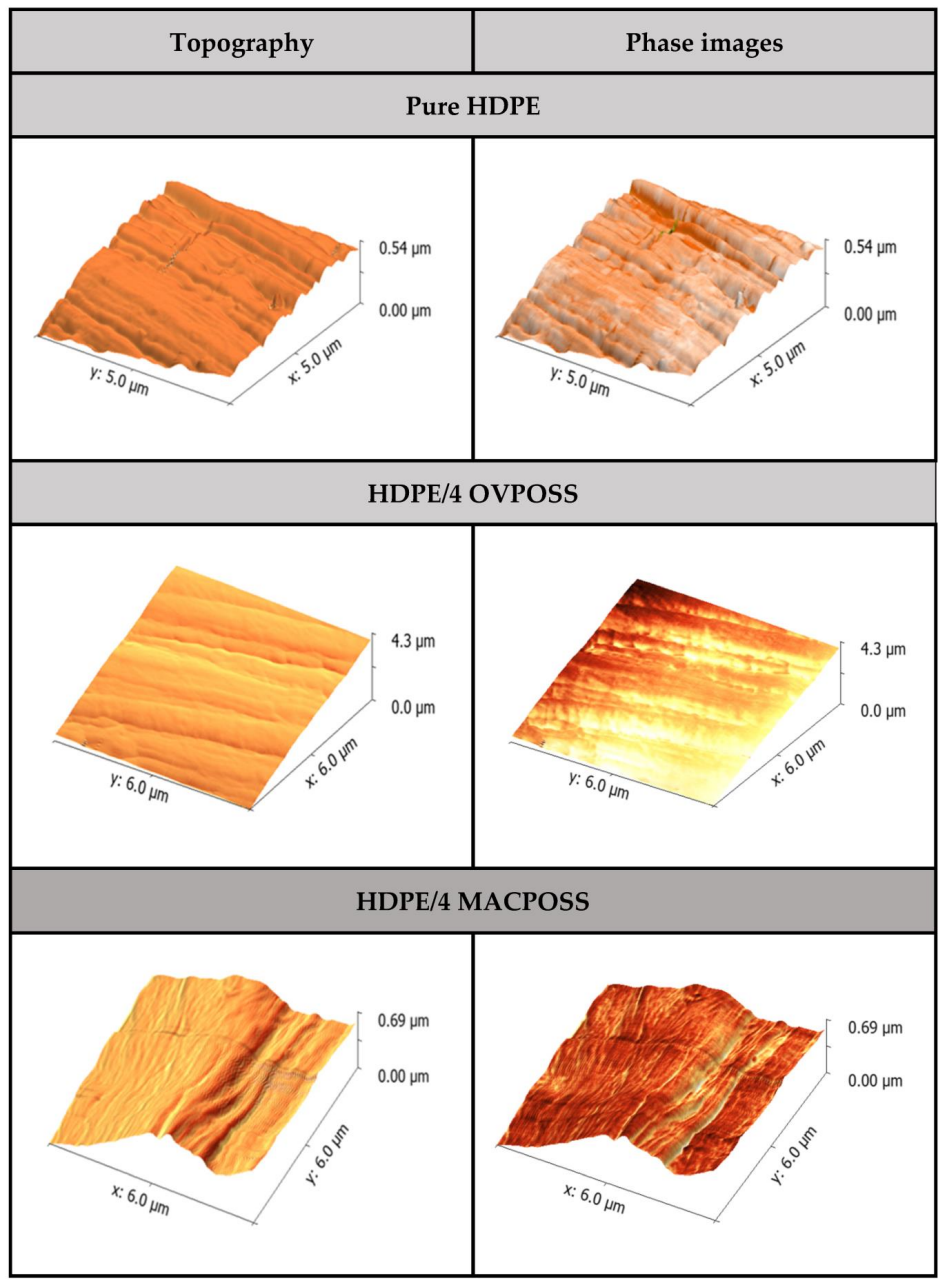
Topography and phase images of the oriented HDPE and HDPE/POSS fibers.

Figure 12 presents AFM topographies and phase images of oriented HDPE/POSS fibers. The images reveal the formation of more microfibril structures in oriented fibers compared to spun fibers. It is well-established that a dominant fibrillar morphological structure emerges when fibers undergo a high draw during orientation. The formation of a shish structure and a concurrent reduction in fiber diameters were observed after orientation. Phase images exhibited a homogeneous dispersion for each type of POSS after orientation. In pure oriented HDPE fibers, bridges between fibers were formed. Additionally, the weaker epitaxial material resembling a kebab structure disappeared with fiber orientation, transforming into a longer chain crystal shish structure.

**Figure 13:**
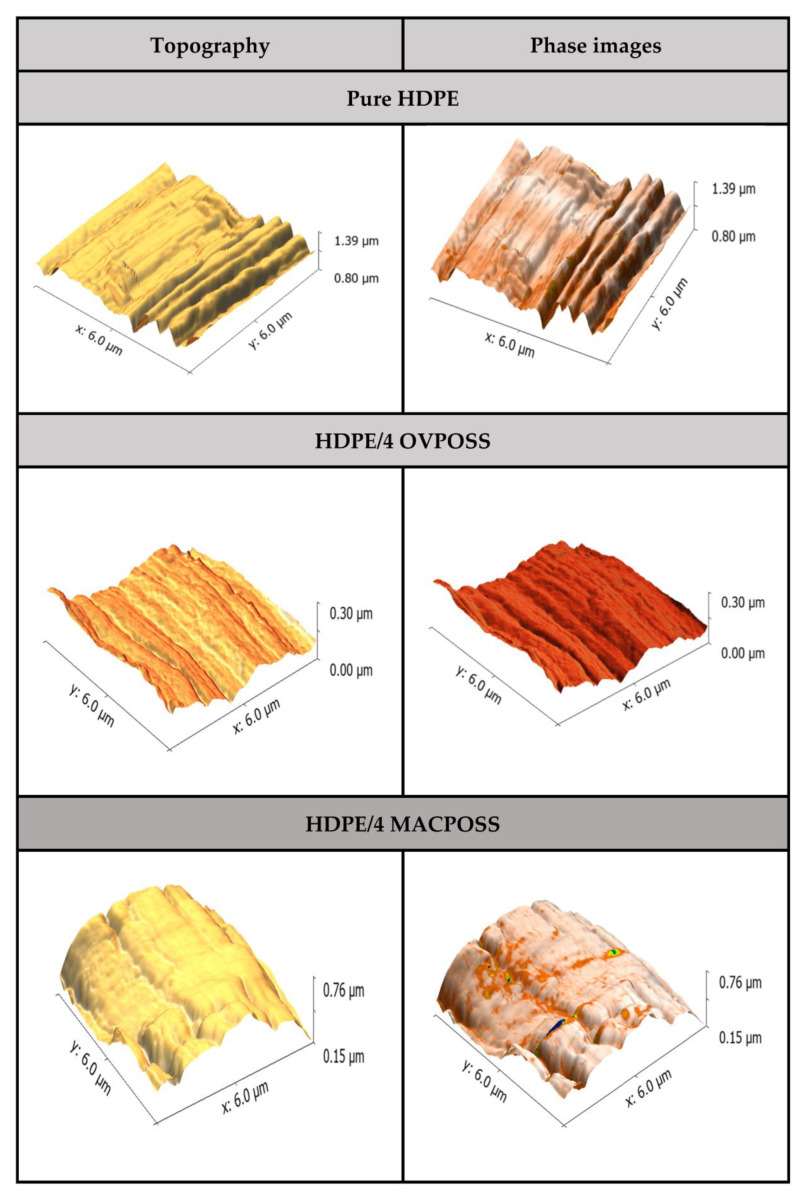
Topography and phase images of the oriented HDPE and HDPE/POSS fibers after UV.

Figure 13 displays AFM topographies and phase images of oriented HDPE/POSS fibers after 10 min of UV exposure. In pure HDPE fibers, an increase in bridging connections between fibrils was noted, accompanied by the observation of gap structures between fibrils. The fibrillar structure remained intact in fibers containing OVPOSS, with an increase in the swelling structures of cross-linked fibers. Microfibril structures with homogeneous diameters were observed. Conversely, in fibers with MACPOSS, an undesired shish-kebab structure formed during cross-linking. Heterogeneous dispersion was observed for MACPOSS, while OVPOSS exhibited a homogeneous dispersion.

### 3.4. Crystal Structure

**Figure 14:**
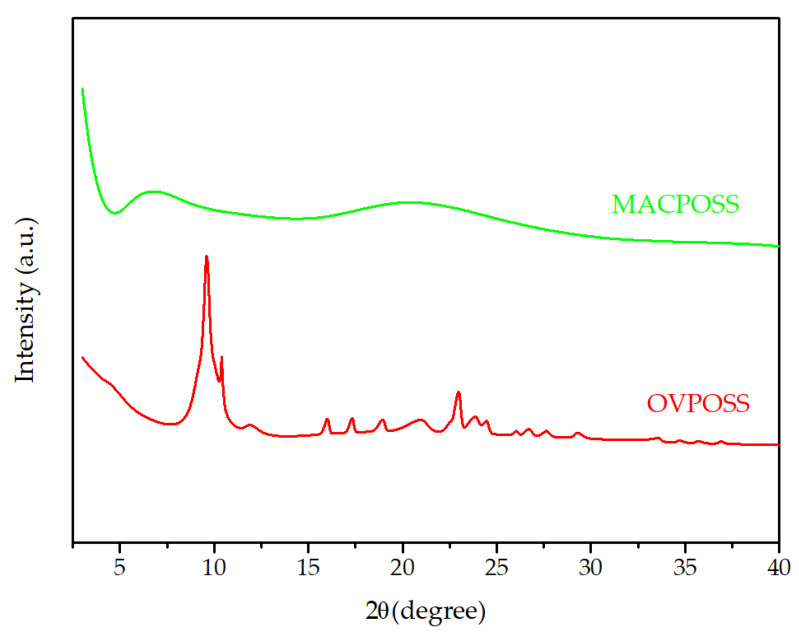
XRD patterns of POSS molecules.

Figure 14 depicts the X-ray diffraction patterns of POSS molecules. OVPOSS exhibits three distinct sharp diffraction peaks at 2θ values of 9.6°, 10.4°, and 23°, indicative of a well-defined crystal structure consistent with existing literature [35]. In contrast, although MACPOSS is liquid in room temperature, it exhibits a peak at 2θ = 6.7°, and a broad halo at 2θ = 19.3° represents a characteristic diffraction peak of Si-O-Si bonds [45]. XRD analyses reveal that OVPOSS possesses a more crystalline structure compared to MACPOSS.

**Figure 15:**
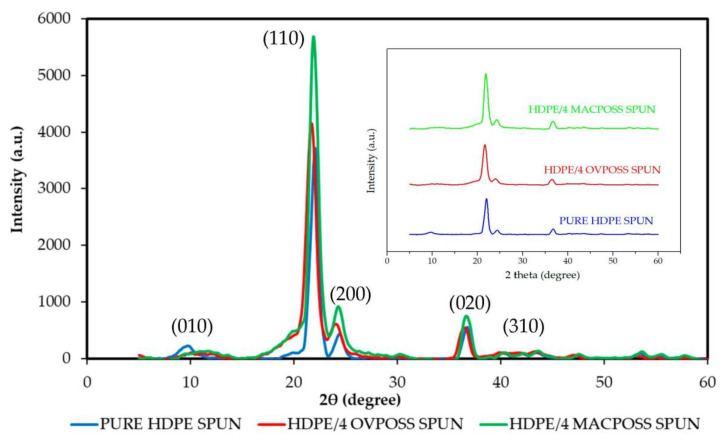
XRD patterns of the spun HDPE and HDPE/POSS fibers.

Figure 15 displays the XRD patterns of spun HDPE and HDPE/POSS fibers. Specifically, the peak observed at 2θ = 21.62° (110) and the presence of two weak diffraction peaks at 2θ = 23.90° (200) and 2θ = 36.03° (020) suggest the orthorhombic crystal lattice structure of HDPE [46,47,48]. Following the orientation of HDPE fibers, changes in the intensity of the (110) and (200) diffraction peaks were observed. Moreover, the (020) diffraction peak diminished, indicating a modification in the orientation of orthorhombic crystals. Furthermore, a distinct (010) diffraction peak indicative of the monoclinic/triclinic HDPE, coexisting with the prevalent orthorhombic form, was prominently identified [49]. The increment in the intensity of the (110) and (200) diffraction peaks became evident with the incorporation of OVPOSS. Enhanced crystallinity values were observed in the spun fibers with the addition of POSS, as detailed in Table 4. Notably, the addition of MACPOSS in spun fibers exhibited a sharper peak compared to OVPOSS. Figure 15 illustrates an increase in intensity in samples containing MACPOSS. This heightened crystallinity in spun HDPE fibers with MACPOSS is attributed to the superior nucleating efficiency of MACPOSS for HDPE. The higher crystallinity in the HDPE fiber containing MACPOSS can be attributed to the increased methylene groups in the lattice structure of MACPOSS, thereby facilitating HDPE crystallization. Conversely, the nucleation effect of OVPOSS for spun HDPE appears less pronounced. Furthermore, it was discerned that the introduction of POSS, regardless of the type, initiated the suppression of the monoclinic crystal lattice structure in melt spinning HDPE fibers.

**Figure 16:**
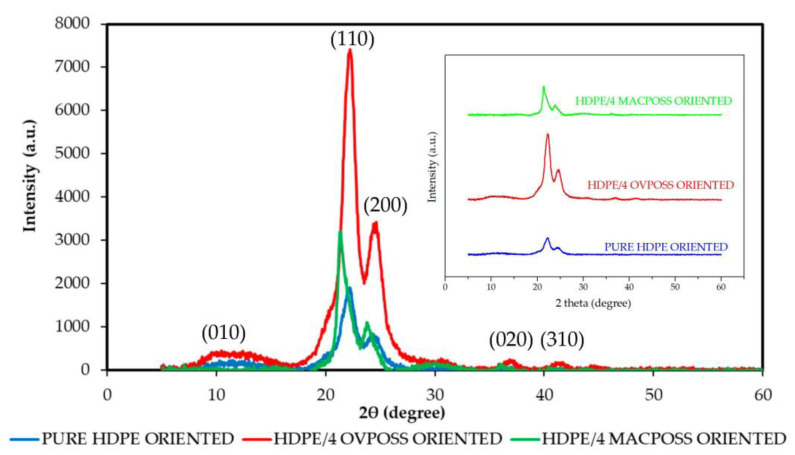
XRD patterns of the oriented HDPE and HDPE/POSS fibers.

The XRD patterns of oriented HDPE and HDPE/POSS fibers are depicted in Figure 16, and the corresponding crystallinity and crystal thickness values for both spun and oriented fibers, derived from XRD patterns, are presented in Table 4. Spun HDPE fibers without POSSs exhibited a crystallinity of 49%, whereas the oriented fibers demonstrated an increase to 55%. It was observed that the XRD peaks sharpened, and the peak widths expanded with fiber orientation during processing, leading to enhanced crystallinity values. This phenomenon was particularly pronounced in the presence of OVPOSS, where the crystallinity reached 63% after orientation. The evident increase in crystallinity values with the orientation and addition of POSS types is attributed to the formation of new crystal structures and heightened crystallization resulting from increased macromolecular chain mobility within the orientational fibers following chain relaxation [50]. Consequently, highly crystalline-oriented fibers with smaller crystal sizes compared to spun fibers were formed. As delineated in Table 4, the crystal thicknesses ranged between 7 and 8 nm after the melt spinning process; however, they decreased by 3–5 nm after the orientation process. This observation supports the formation of smaller crystals after the orientation of fibers, regardless of the fiber type.

It was revealed that the weaker epitaxial material, characterized by a kebab structure, undergoes a transformation into an extended chain crystal shish structure subsequent to the spinning of fibers. Under increased stress conditions, amorphous layers initially undergo elongation, initiating the sliding of the highly oriented lamellae and consequently leading to a rapid reduction in their lateral dimensions. Further escalation of applied stress induces fragmentation of the lamellae, fostering a stress-induced recrystallization process, culminating in the formation of shish crystals. The incremental addition of adjacent chains to the existing shish crystals is observed, with structures situated away from the shish crystals undergoing a metamorphic process to manifest as new shish crystals. Consequently, the quantity of shish crystals increased with the applied strain. Notably, a swift transition is discerned from a spherulitic structure to a row-nucleated structure in spun fibers. Under low-stress conditions, the row structure is characterized by lamellae emanating from fibril nuclei and oriented perpendicular to the fiber axis. This lamellar spherulite adopts a bent configuration reminiscent of the bending observed in the radial direction for lamellar crystals originating from the central region. Conversely, at elevated draw speeds and spin line stresses, the lamellar crystals constituting the row structure exhibit diminished bending compared to their counterparts under lower stress conditions. The phenomenon of strain-induced fibril nucleation is identified as a catalyst for crystallization at temperatures significantly exceeding those associated with crystallization in an inert melt subjected to similar cooling rates. Consequently, crystallization under these conditions results in reduced interlamellar spaces, thereby establishing the dominance of a fibrillar morphology in a highly spun fiber.

During the orientation process, samples having OVPOSS showed a discernible increment in the peak density of the crystalline structure. Peaks were located at 2θ = 11.86° (010) and 2θ = 40.79° (310), conforming to polyethylene fibers aligned in a manner analogous to those documented in the existing literature. Notably, the intensity of these peaks exhibited an enhanced prominence in the presence of OVPOSS, signifying the emergence and coexistence of monoclinic/triclinic crystals alongside the prevalent orthorhombic configuration. Contrary to expectations, the orthorhombic crystal peak at 2θ = 36.03° (020) in the spun fiber persisted after orientation, and its intensity underwent a shift to 2θ = 30° peaks. Furthermore, OVPOSS manifested the most substantial amplification in the crystalline peak intensity following the orientation process, while fibers with MACPOSS exhibited a comparatively minor influence on crystalline peak intensity during orientation. In contrast to spun HDPE fibers, oriented HDPE fibers incorporating OVPOSS showcased markedly elevated crystallinity values, indicative of OVPOSS contributing to the conformational order of HDPE during the orientation process. This phenomenon is ascribed to the chemical compatibility between OVPOSS and HDPE. Surprisingly, the incompatibility between the polar nature of MACPOSS and the nonpolar character of HDPE also resulted in an increment in the crystallinity of HDPE subsequent to the orientation process. Moreover, its crystallinity is still higher than that of spun and oriented HDPE fiber.

**Figure 17:**
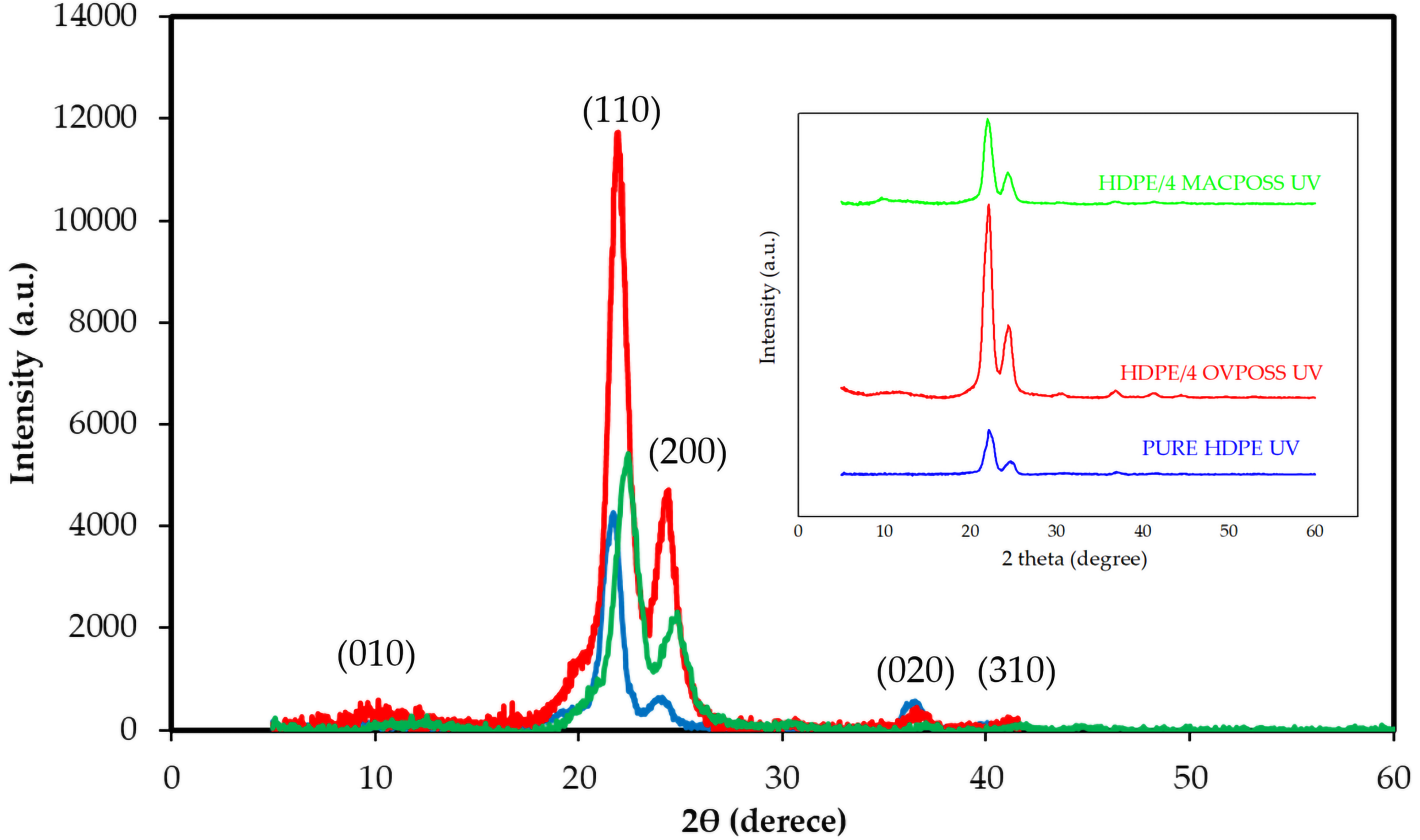
XRD patterns of the oriented HDPE and HDPE/POSS fibers after UV.

The XRD patterns of UV cross-linked oriented HDPE/POSS fibers are illustrated in Figure 17. Notably, a discernible augmentation in intensity is evident after cross-linking. Specifically, fibers incorporating OVPOSS exhibited a more pronounced crystalline structure in comparison to those incorporating MACPOSS. The 58% crystallinity of cross-linked pure HDPE was increased by 65% with the addition of OVPOSS. Similarly, the addition of MACPOSS resulted in an enhanced crystallinity value, reaching 61%. This heightened crystallinity is attributed to the phenomenon of secondary crystallization [51], instigated by the formation of shorter segments with increased mobility during the degradation process [52]. The rupture of binding molecules as they traverse amorphous sections, coupled with the subsequent rearrangement of the liberated segments, potentially contributes to an augmentation in lamellar thicknesses within crystalline regions, thereby accounting for the observed shift in crystallinity. This process substantiates the alignment of incompletely crystallized chains during the manufacturing phase, fostering an increase in overall crystallinity [43].

### 3.5. Thermal Properties

The thermal stability of both HDPE and HDPE/POSS fibers was systematically assessed through a thermogravimetric analysis (TGA), and the resulting dataset from TGA curves is presented in Table 5. TGA curves and weight derivatives of the spun, oriented, and UV-irradiated HDPE and HDPE/POSS fibers are shown in Supplementary Materials (Figures S1–S6). Moreover, T_d,5_, T_d,10_, and T_d,max_ represent the decomposition temperatures of samples where 5%, 10%, and maximum weight loss occur. As seen from Figures S1, S3 and S5, all the samples exhibited one-step degradation behavior.

Onset degradation temperatures of spun HDPE fibers and spun HDPE/MACPOSS fibers yielded similar values. However, the onset degradation temperature of the HDPE spun fiber shifted to higher values with the addition of 4 phr OVPOSS, which was due to the significant improvement in crystallinity in the presence of OVPOSS. Orientation and UV irradiation did not affect the onset degradation temperatures of all samples, as seen in Table 5. On the other hand, T_d,max_ values of spun, oriented, and UV-irradiated samples, including POSS nanoparticles, regardless of the POSS type, showed higher values than that of HDPE samples without POSSs. The improvement in the thermal stability of all HDPE fibers in the presence of POSS molecules can be attributed to the increased crystallinity. Moreover, POSS nanoparticles can act as a physical barrier via their inorganic Si-O cage-like skeleton for the HDPE matrix that prevents heat flux. As expected, the residue percent increased after the addition of POSS nanoparticles to the HDPE since POSS molecules have inorganic Si atoms in their structures.

**Figure 18:**
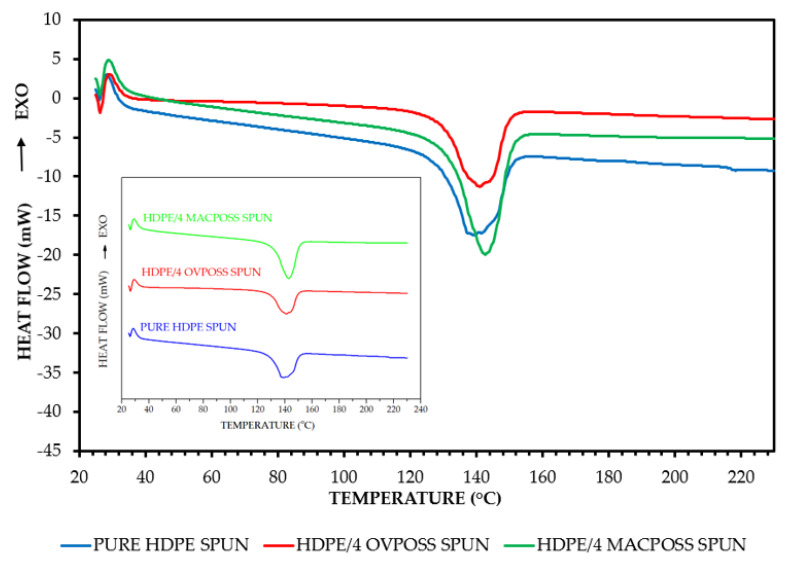
DSC thermograms of spun HDPE and HDPE/POSS fibers.

**Figure 19:**
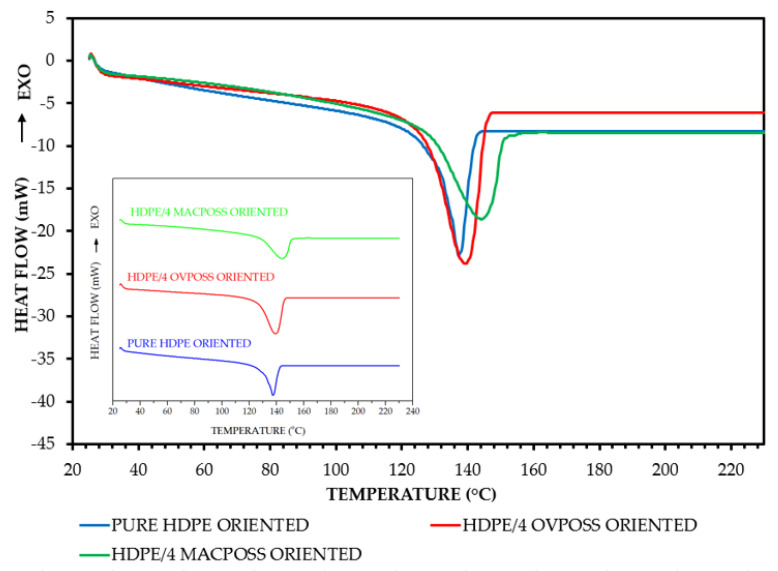
DSC thermograms of oriented HDPE and HDPE/POSS fibers.

The DSC thermograms depicting the melting behavior of spun HDPE and HDPE/POSS fibers are illustrated in Figure 18. Evidently, all fibers exhibited a distinct melting endotherm, with comparable melting temperatures. Notably, the introduction of POSS nanoparticles into the HDPE matrix did not exert discernible influence on the melting behavior of HDPE. However, there was a slight improvement in melting enthalpy of the HDPE spun fiber with the incorporation of POSS. Specifically, the addition of POSS to the pure spun HDPE fiber, characterized by a crystallinity value of 49%, led to an increase in percent crystallinity to values ranging between 50% and 53% (Table 6). The most substantial increment in crystallinity among spun fibers was observed in the case of HDPE/MACPOSS. The DSC thermogram of oriented HDPE and HDPE/POSS fibers, depicted in Figure 19, revealed enlarged melting endotherm peaks attributed to the orientation-induced alterations in crystal morphologies. Furthermore, a significant increase in percent crystallinity values was evident after orientation, owing to the formation of new crystals. Additionally, the incorporation of POSS contributed to the improvement of crystallinity in oriented fibers, with the highest increase observed with the addition of OVPOSS. These findings align with XRD results, wherein the homogenous dispersion of OVPOSS in HDPE facilitated crystal formation during the orientation process.

**Figure 20:**
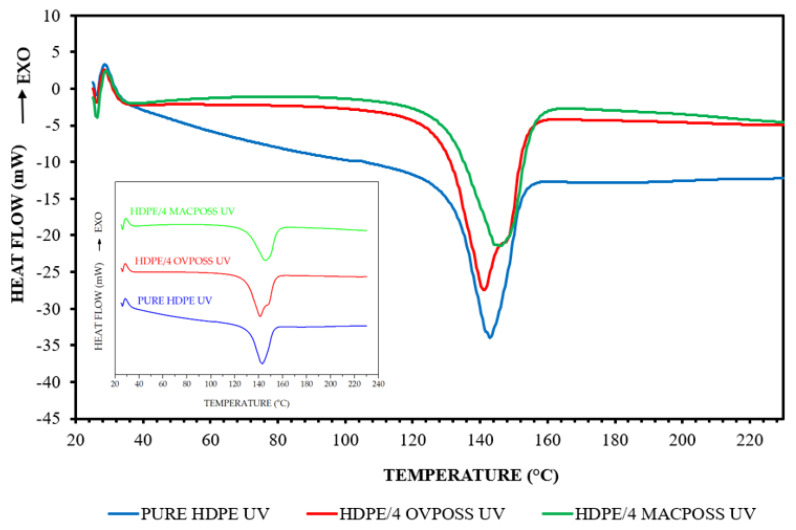
DSC thermograms of oriented HDPE and HDPE/POSS fibers after UV irradiation.

DSC thermograms of samples after UV cross-linking are shown in Figure 20. As seen in Figure 20, melting endotherms were found to be more broadened after UV cross-linking. The melting enthalpy of oriented HDPE fibers did not change after UV irradiation. However, decrements in melting enthalpy values were observed for UV-irradiated HDPE/OVPOSS and HDPE/MACPOSS fibers. On the other hand, the percent crystallinity of oriented HDPE fibers with and without POSS molecules exhibited nearly similar values after the UV irradiation process. These findings can be attributed to less perfect crystal formation in the presence of POSS nanoparticles. However, the percent crystallinity values of UV-irradiated HDPE and HDPE/POSS fibers are still higher than those of spun counterparts.

**Figure 21:**
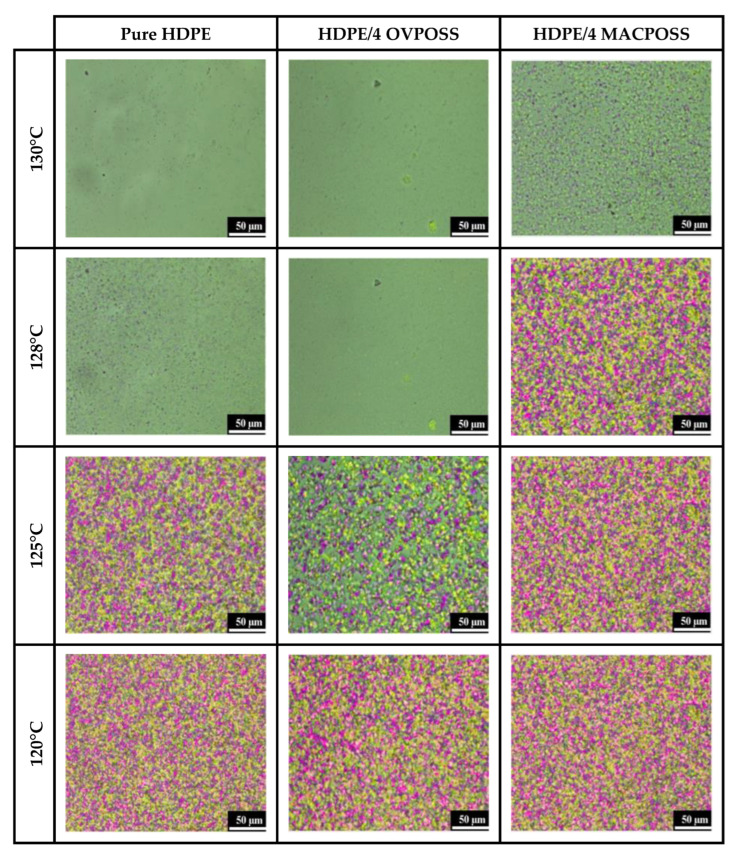
POM images of spun HDPE and HDPE/POSS compounds.

POM analyses were conducted to investigate the melt crystallization behavior of pure HDPE and HDPE/POSS compounds during the cooling process. This examination of crystalline properties was imperative due to the loss of the fiber form in the samples upon melting. In coherence with the data acquired from the DSC results, POM images were taken within the temperature range of 130 °C to 120 °C. The POM images of spun HDPE/POSS compounds are presented in Figure 21. Notably, in spun pure HDPE compounds, the initiation of embryo crystal formation was observed at 128 °C, followed by the completion of smaller crystal nuclei at 125 °C. Conversely, with the addition of OVPOSS, similar crystal development completed nearly at 125 °C, with no discernible nucleating effect for the HDPE spun compounds. In contrast, the crystallization onset temperature shifted to higher temperatures with the addition of MACPOSS. MACPOSS exhibited the promotion of embryo crystals even at 130 °C, and an enhanced spherulite density was determined. Furthermore, the completion of crystals was observed at 128 °C in the presence of MACPOSS. These observations can be attributed to the nucleation effect of MACPOSS on spun HDPE compounds, facilitated by its flexible alkyl groups enhancing nucleation ability.

**Figure 22:**
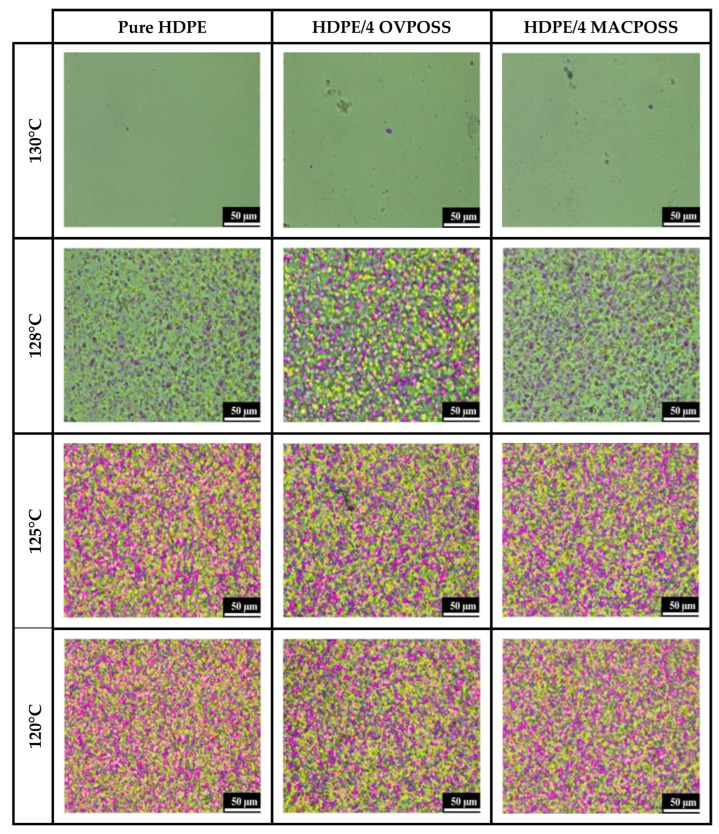
POM images of oriented HDPE and HDPE/POSS compounds.

POM images of oriented HDPE/POSS compounds are shown in Figure 22. It was observed that the crystal morphology changed after orientation. As seen, the crystal formation temperature shifted to lower temperatures with orientation and nuclei began to form at 128 °C. Afterward, it was seen that the crystal nuclei continue to form. The temperature at which embryo crystals begin to form could not be clearly observed. The nucleation effect of OVPOSS was found to be similar to other POSS types after orientation. It can be concluded that OVPOSS has a nucleating agent effect as it supports the formation of crystal nuclei. Similar crystal formation was observed in oriented pure HDPE and MACPOSS-added compounds. In addition, similar micron-sized crystals began to form at 125 °C with the addition of OVPOSS and MACPOSS. As a general conclusion, all POSS molecules acted as nucleating agents for HDPE compounds.

**Figure 23:**
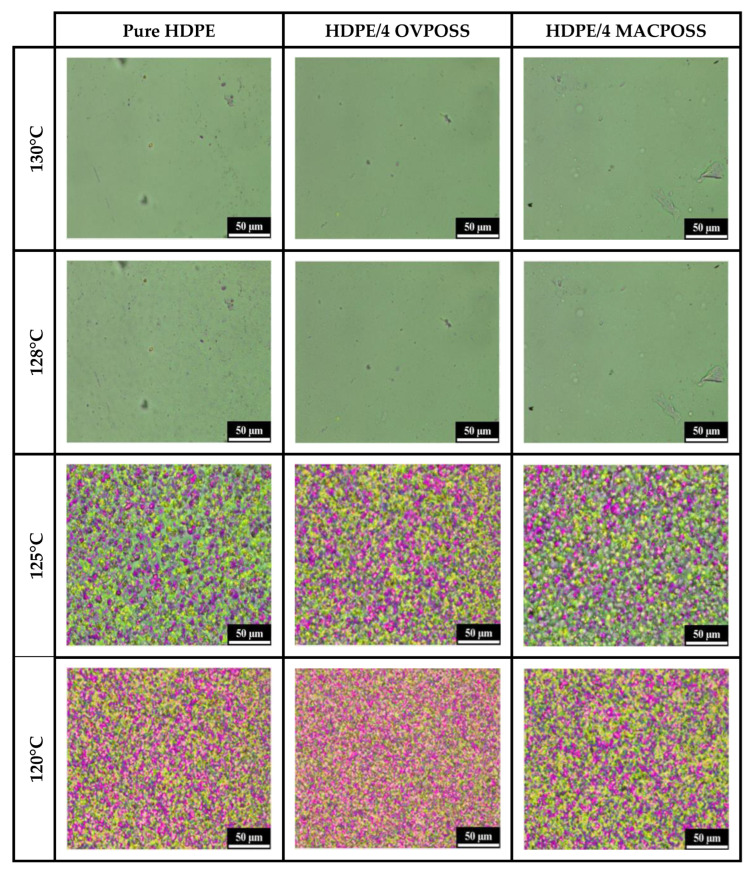
POM images of oriented HDPE and HDPE/POSS compounds after UV.

POM images of cross-linking with UV HDPE/POSS compounds are shown in Figure 23. No crystal formation was observed at 128 °C since crystal formation was prevented after cross-linking. Crystal formation was observed at 125 °C. It was observed that the crystallization was completed at 120 °C.

## 4. Conclusions

In this investigation, an environmentally friendly and cost-effective approach was employed to fabricate high-performance, oriented, and UV cross-linked HDPE spun fibers in the presence of distinct POSS molecules featuring reactive functional groups. Effects of POSS molecules having vinyl and methacryl functional groups on the chemical, mechanical, thermal, morphological, and crystallographic properties of HDPE fibers were comprehensively examined. The modulus of oriented fibers with OVPOSS experienced a notable enhancement of 60% compared to oriented HDPE fibers without POSS. Subsequent UV cross-linking resulted in additional improvements in mechanical and thermal properties. Upon the orientation and UV cross-linking of HDPE fibers in the presence of both OVPOSS and MACPOSS, the tensile strength value was significantly enhanced. Among the POSS nanoparticles, the level of dispersion of OVPOSS was found to be homogeneous due to its chemical compatibility with HDPE. On the other hand, the agglomeration of MACPOSS caused lower mechanical properties; however, it is still higher than pure HDPE fibers. Observations indicated a reduction in fiber diameters to approximately 90 µm through the orientation process. XRD and DSC analyses revealed an increase in percent crystallinity values with the inclusion of POSS types, coupled with a reduction in crystal thicknesses due to orientation. Thermal property assessments underscored the positive influence of both POSS addition and fiber orientation on thermal stability. Upon comprehensive evaluation, the incorporation of POSS nanoparticles notably improved the overall performance of the HDPE fiber. This research is considered a pivotal advancement toward the production of thinner, high-performance fibers with potential applications in composites, protective clothing, and diverse industrial sectors.

## Figures and Tables

**Table 1:** Compositions of the fibers.

Sample Name	HDPE Content (phr)	POSS Content (phr)
Pure HDPE	100	-
HDPE/4 OVPOSS	100	4
HDPE/4 MACPOSS	100	4

**Table 2:** Mechanical properties of spun, oriented, and UV-irradiated HDPE and HDPE/POSS fibers.

Sample Name		Tensile Strength (MPa)	Elongation at Break (%)	Modulus (GPa)	Energy at Break (J)
Spun	Pure HDPE	24 ± 2	1007 ± 113	1.4 ± 0.1	0.315 ± 0.02
	HDPE/4 OVPOSS	41 ± 4	797 ± 48	1.9 ± 0.2	0.335 ± 0.01
	HDPE/4 MACPOSS	25 ± 3	891 ± 133	1.5 ± 0.2	0.097 ± 0.03
Oriented	Pure HDPE	265 ± 6	83 ± 23	1.5 ± 0.2	0.032 ± 0.01
	HDPE/4 OVPOSS	279 ± 5	47 ± 8	2.4 ± 0.2	0.023 ± 0.02
	HDPE/4 MACPOSS	275 ± 3	64 ± 17	1.6 ± 0.2	0.024 ± 0.01
After UV	Pure HDPE	264 ± 4	62 ± 14	2.2 ± 0.3	0.026 ± 0.03
	HDPE/4 OVPOSS	293 ± 5	44 ± 20	2.8 ± 0.2	0.036 ± 0.04
	HDPE/4 MACPOSS	283 ± 2	63 ± 22	2.5 ± 0.2	0.030 ± 0.02

**Table 3:** Average fiber diameters of the spun, oriented, and UV-irradiated HDPE and HDPE/POSS fibers.

Sample Name	Average Fiber Diameters (µm)		
	Spun	Oriented	After UV
Pure HDPE	237 ± 22	92 ± 3	81 ± 19
HDPE/4 OVPOSS	237 ± 9	78 ± 8	78 ± 9
HDPE/4 MACPOSS	346 ± 27	93 ± 11	90 ± 11

**Table 4:** Crystallinity and crystal thickness of the spun, oriented, and UV cross-linked HDPE and HDPE/POSS fibers.

**Sample Name**	**Crystallinity (%)**			**Crystal Thickness (nm)**		
	**Spun**	**Oriented**	**After UV**	**Spun**	**Oriented**	**After UV**
Pure HDPE	49	55	58	7.1	3.7	6.3
HDPE/4 OVPOSS	52	63	65	8.1	5.5	5.0
HDPE/4 MACPOSS	53	60	61	4.3	3.7	5.1

**Table 5:** Decomposition temperatures and residue percentage of the spun, oriented, and after-UV HDPE and HDPE/POSS fibers from the TGA results.

Sample Name		T_d,5_ (°C)	T_d,10_ (°C)	T_d,max_ (°C)	Residue Percentage (%)
Spun	Pure HDPE	445.6	456.7	473.7	2.6
	HDPE/4 OVPOSS	450.8	456.7	475.2	3.7
	HDPE/4 MACPOSS	445.6	457.1	475.9	2.7
Oriented	Pure HDPE	450.8	456.7	475.9	2.4
	HDPE/4 OVPOSS	450.8	456.7	477.1	5.2
	HDPE/4 MACPOSS	450.8	456.7	478.7	4.8
After UV	Pure HDPE	450.8	457.1	476.7	3.3
	HDPE/4 OVPOSS	450.8	457.1	480.4	4.1
	HDPE/4 MACPOSS	450.8	456.7	478.8	4.1

**Table 6:** Thermal properties and percent crystallinity of the spun, oriented, and UV-irradiated HDPE and HDPE/POSS fibers.

Sample Name		T_m,onset_ (°C)	T_m,peak_ (°C)	T_m,endset_ (°C)	ΔH_m_ (J/g)	X_c_ (%)
Spun	Pure HDPE	129	140	153	145.5	49.6
	HDPE/4 OVPOSS	128	143	150	147.8	50.4
	HDPE/4 MACPOSS	127	146	148	154.4	52.7
Oriented	Pure HDPE	132	142	150	158.5	56.3
	HDPE/4 OVPOSS	130	140	149	172.4	60.6
	HDPE/4 MACPOSS	131	145	148	167.0	59.4
After UV	Pure HDPE	130	139	150	159.8	54.5
	HDPE/4 OVPOSS	131	141	152	169.4	60.2
	HDPE/4 MACPOSS	130	145	155	154.4	54.9

## Data Availability

The data presented in this study are available on request from the corresponding author.

## Supplementary Materials

The following supporting information can be downloaded at: https://www.mdpi.com/article/10.3390/nano13243131/s1, Figure S1: TGA curves of the spun HDPE and HDPE/POSS fibers; Figure S2: Derivative weight of the spun HDPE and HDPE/POSS fibers; Figure S3: TGA curves of the oriented HDPE and HDPE/POSS fibers; Figure S4: Derivative weight of the oriented HDPE and HDPE/POSS fibers; Figure S5: TGA curves of the oriented HDPE and HDPE/POSS fibers after UV; Figure S6: Derivative weight of the oriented HDPE and HDPE/POSS fibers after UV.
