# Supplementary material for: Processing and Characterization of UV Irradiated HDPE/POSS Fibers
Source: Nanomaterials (Basel). 2023 Dec 13;13(24):3131. doi: 10.3390/nano13243131 (PMC10745762; doi:10.3390/nano13243131)
Supplement: Supplementary file 1 [file nanomaterials-13-03131-s001.zip › nanomaterials-2711237-supplementary.pdf]

Supplementary Material

Processing and Characterization of UV Irradiated HDPE/POSS Fibers

Ezgi Biçer [1], Mehmet Kodal [1,2,*] and Güralp Özkoç [2,3,4]

[1] Department of Chemical Engineering, Kocaeli University, 41001, Kocaeli, Turkey; ezgibicer39@gmail.com (E.B.); mehmet.kodal@kocaeli.edu.tr (M.K.)
[2] Nanotechnology Research and Application Center, Sabancı University, 34956, Istanbul, Turkey; guralp.ozkoc@istinye.edu.tr
[3] Department of Chemistry, Istinye University, 34396, Istanbul, Turkey
[4] Xplore Instruments B.V., 6135 KT, Sittard, The Netherlands
* Correspondence: mehmet.kodal@kocaeli.edu.tr, Tel.: +902623033540

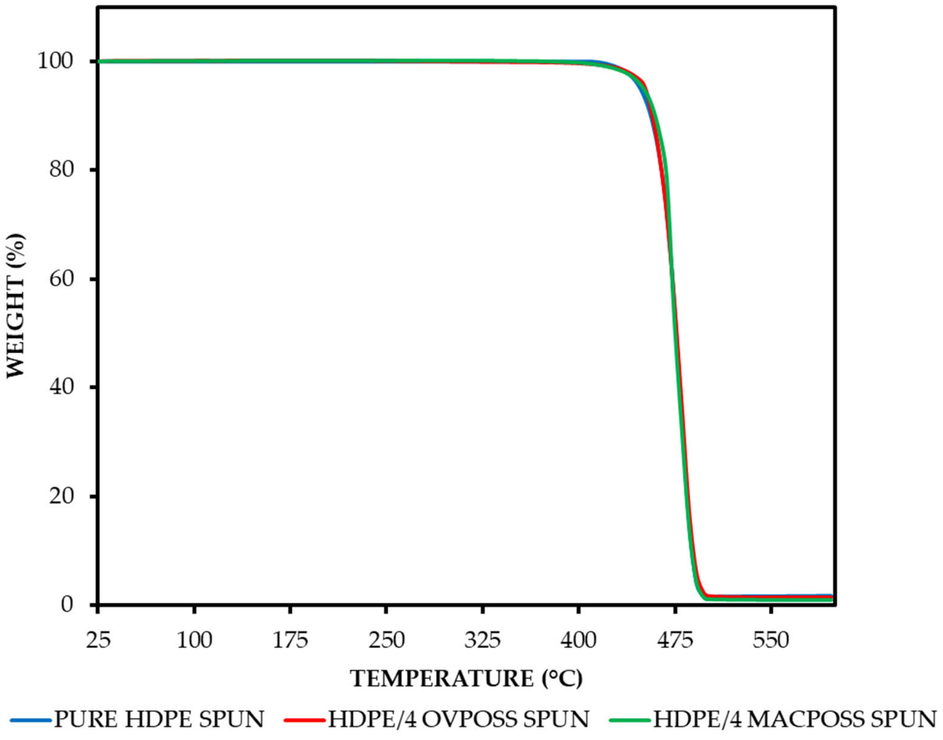

Figure S1. TGA curves of the spun HDPE and HDPE/POSS fibers.

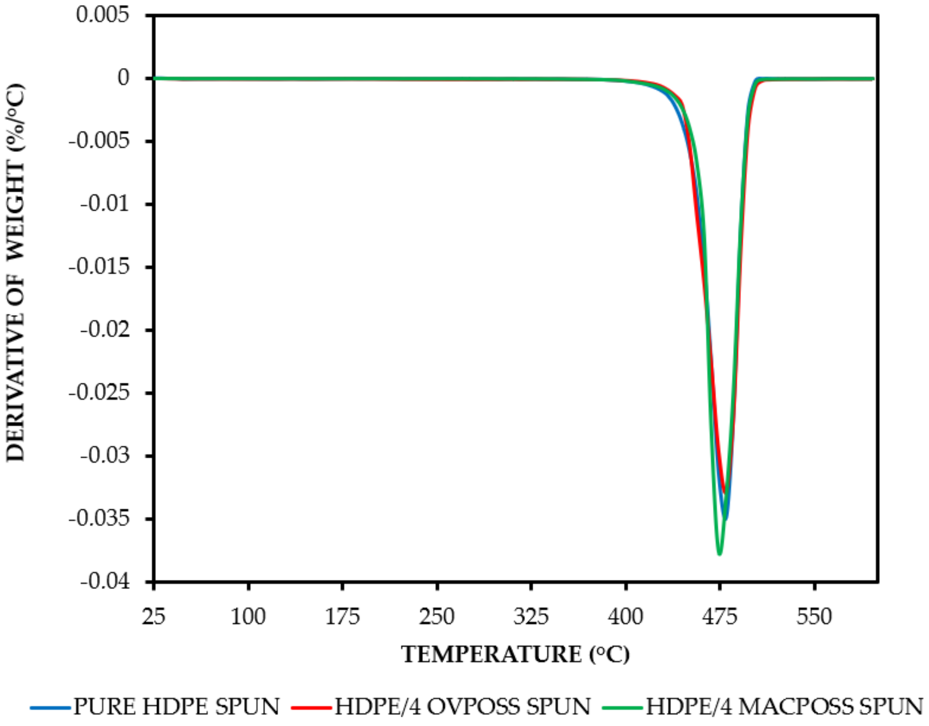

Figure S2. Derivative weight of the spun HDPE and HDPE/POSS fibers.

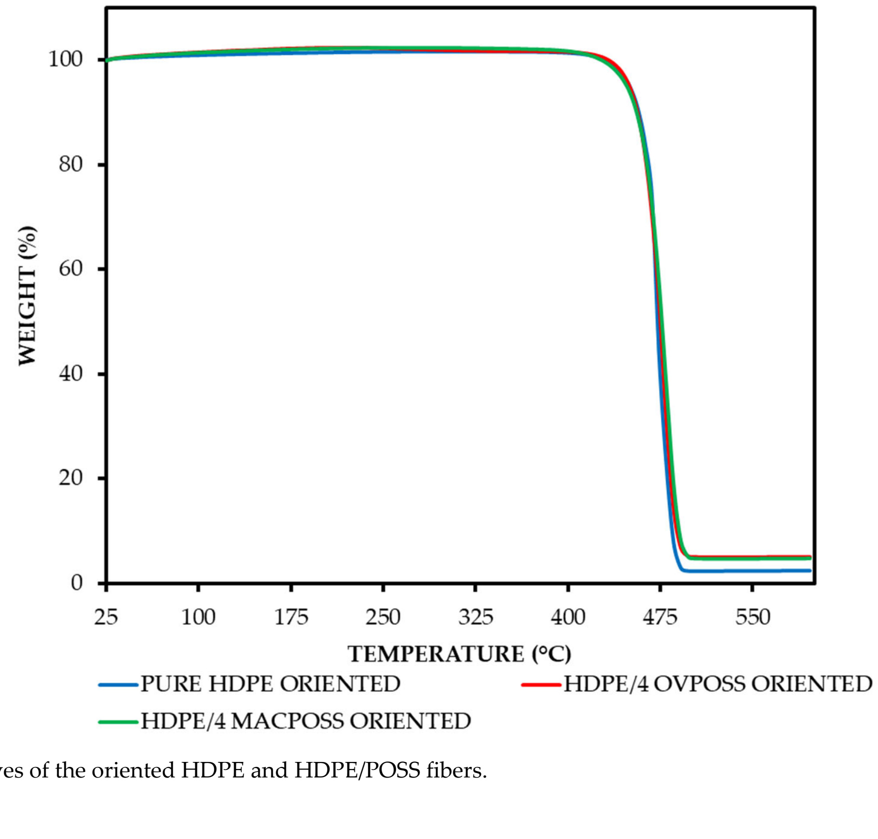

Figure S3. TGA curves of the oriented HDPE and HDPE/POSS fibers.

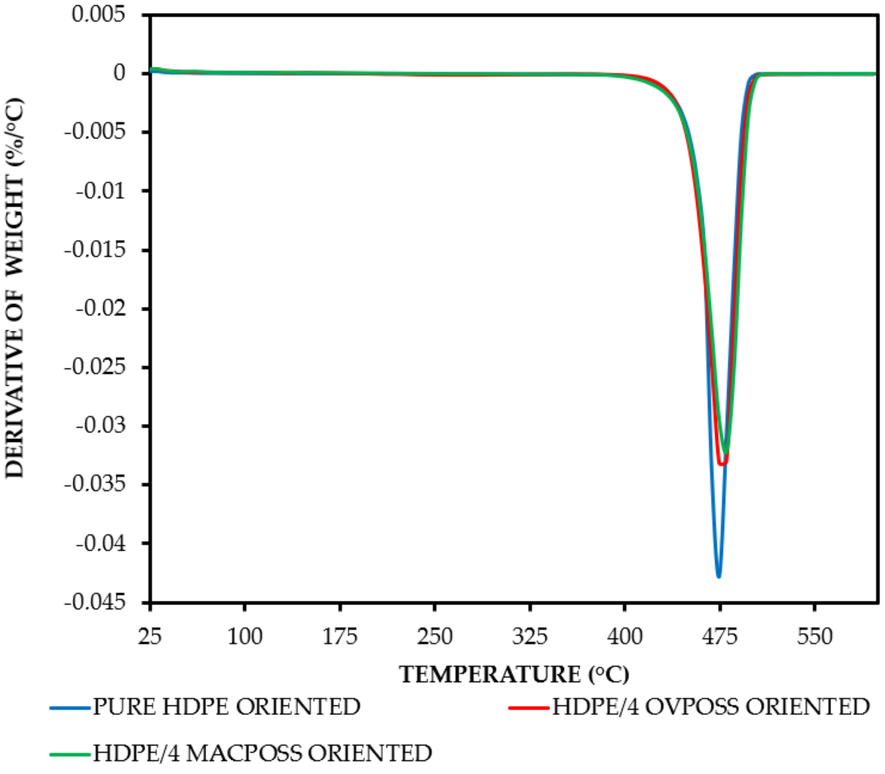

Figure S4. Derivative weight of the oriented HDPE and HDPE/POSS fibers.

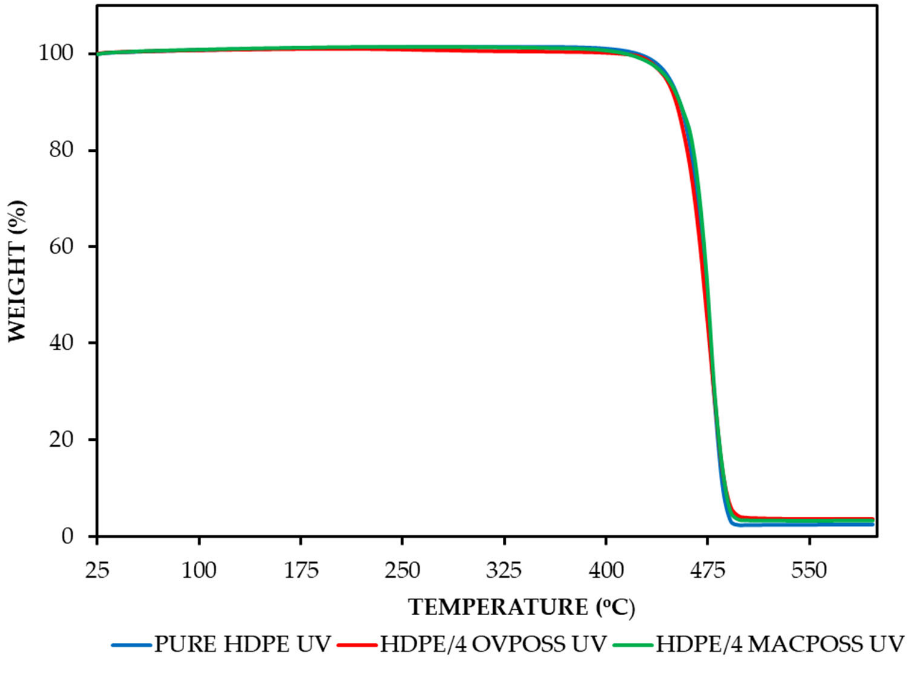

Figure S5. TGA curves of the oriented HDPE and HDPE/POSS fibers after UV.

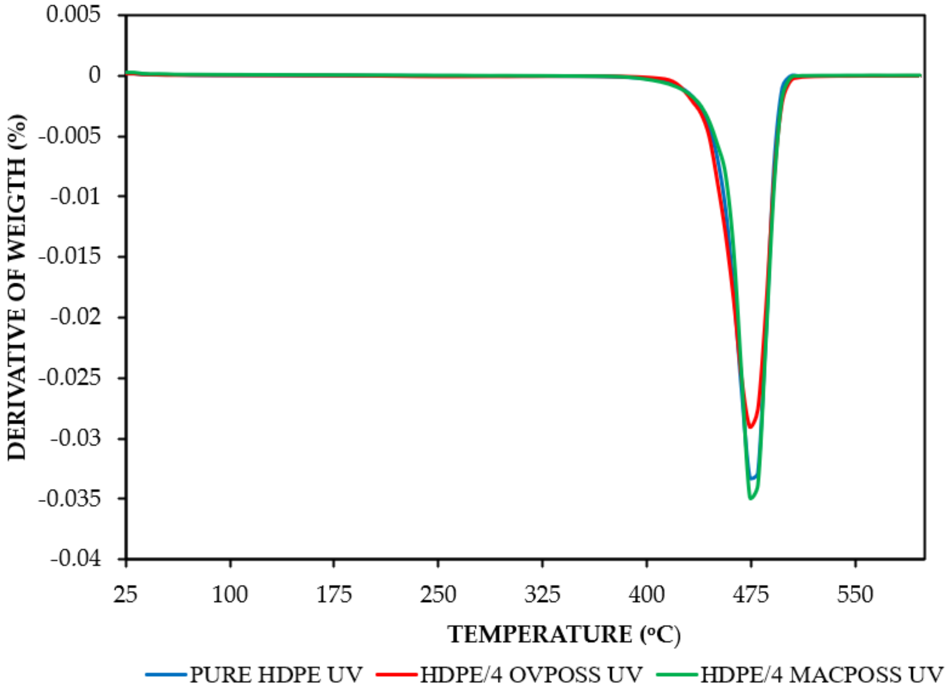

Figure S6. Derivative weight of the oriented HDPE and HDPE/POSS fibers after UV.
